# The Associations between Multiple Essential Metal(loid)s and Gut Microbiota in Chinese Community-Dwelling Older Adults

**DOI:** 10.3390/nu15051137

**Published:** 2023-02-24

**Authors:** Jianghui Zhang, Yuan Wang, Guimei Chen, Hongli Wang, Liang Sun, Dongmei Zhang, Fangbiao Tao, Zhihua Zhang, Linsheng Yang

**Affiliations:** 1 Department of Epidemiology and Biostatistics, School of Public Health, Anhui Medical University, Hefei 230032, China; 2 Anhui Provincial Key Laboratory of Population Health and Aristogenics, Hefei 230032, China; 3 School of Health Services Management, Anhui Medical University, Hefei 230032, China; 4 Fuyang Center for Disease Control and Prevention, Fuyang 236069, China

**Keywords:** essential metal(loid)s, gut microbiota, 16S rRNA gene, BKMR, epidemiology

## Abstract

Several experimental studies have suggested that individual essential metal(loid)s (EMs) could regulate the gut microbiota. However, human studies assessing the associations between EMs and gut microbiota are limited. This study aimed to examine the associations of individual and multiple EMs with the compositions of the gut microbiota in older adults. A total of 270 Chinese community-dwelling people over 60 years old were included in this study. Urinary concentrations of selected EMs, including vanadium (V), cobalt (Co), selenium (Se), strontium (Sr), magnesium (Mg), calcium (Ca), and molybdenum (Mo), were examined by inductively coupled plasma mass spectrometry. The gut microbiome was assessed using the 16S rRNA gene sequencing analysis. The zero-inflated probabilistic principal components analysis PCA (ZIPPCA) model was performed to denoise substantial noise in microbiome data. Linear regression and the Bayesian Kernel Machine Regression (BKMR) models were utilized to determine the associations between urine EMs and gut microbiota. No significant association between urine EMs and gut microbiota was found in the total sample, whereas some significant associations were found in subgroup analyses: Co was negatively associated with the microbial Shannon (*β* = −0.072, *p* < 0.05) and the inverse-Simpson (*β* = −0.045, *p* < 0.05) indices among urban older adults; Ca (*R*^2^ = 0.035) and Sr (*R*^2^ = 0.023) exhibited significant associations with the altercations of beta diversity in females, while V (*R*^2^ = 0.095) showed a significant association with altercations of beta diversity in those who often drank. Furthermore, the associations between partial EMs and specific bacterial taxa were also found: the negative and linear associations of Mo with Tenericutes, Sr with Bacteroidales, and Ca with Enterobacteriaceae and Lachnospiraceae, and a positive and linear association of Sr with Bifidobacteriales were found. Our findings suggested that EMs may play an important role in maintaining the steady status of gut microbiota. Prospective studies are needed to replicate these findings.

## 1. Introduction

The human gut microbiome comprises 10 trillion diverse symbionts (50 bacterial phyla and about 100–1000 bacterial species) [1], which maintain a close symbiotic relationship with the human body [2]. The gut microbiota remains relatively stable during adulthood, but its compositions are constantly changed during infancy and old age [3]. The alterations of gut microbiota with age are characterized by progressive decreases of overall diversity, core microbiota, and other health-associated bacteria, ultimately leading to gut microbiota dysbiosis [4,5,6,7,8]. Gut dysbiosis may trigger the innate immune response and result in chronic low-grade inflammation, which consists of the basic mechanisms underlying age-related diseases, such as atherosclerosis, diabetes, hypertension, cancer, Alzheimer’s diseases, et al. [9]. Therefore, an in-depth exploration of modifiable risk factors for gut dysbiosis in older adults is necessary to promote healthy aging.

Of the modifiable factors, diet emerges as one of the pivotal determinants. Nutrients in food can directly interact with microorganisms and reshape the gut microbiota [10], which may be especially true for older adults. The physical changes with age such as reduction in dentition, impairment of taste and olfaction, and an increased level of satiation may decrease food intakes and limit the choices for food diversity, which in turn cause malnutrition and alterations of gut microbiota in older adults [11,12]. Essential metal(loid)s (EMs), such as vanadium (V), cobalt (Co), selenium (Se), strontium (Sr), magnesium (Mg), calcium (Ca), and molybdenum (Mo) et al., are essential for biological functions. EMs cannot be produced endogenously and mainly rely on dietary intake [13]. As a result, older adults are more prone to deficiencies of EMs due to their reduced food intake. A recent systematic review found that there were 31% of women and 49% of men living with zinc deficiencies and 49% of women and 37% of men living with selenium deficiencies in community-dwelling older adults [14]. In recent years, an emerging biologic pathway by which EMs play biological functions is that EMs could reshape and modulate the gut microbiota [15]. For instance, a previous animal study found that calcium (Ca) had a prebiotic-like effect. That is, Ca supplementation can increase the abundance of *Bifidobacterium* and *Bacteroides* [16]. Another animal study also showed that both low and high Ca concentrations led to changes in microbial composition in mice, and the high level of Ca supplement even significantly decreased plasma biomarkers for the metabolic disorder [17]. In addition, dietary Se supplementation at a dose range of 0.1 μg/g through 2.25 μg/g in mice could increase microbial diversity [18]. A similar study indicated that the Se supplement had a beneficial impact on the proliferation of *lactic acid bacteria* and other beneficial bacteria such as *Bacteroides*, *Prevotella*, and *Roseburia* [19]. Despite experimental studies that have provided convincing evidence, epidemiological studies on the association between EMs and the gut microbiota were scarce and did not yield consistent findings [20,21]. The reasons for these mixed results remained unclear. One possibility is that single EMs may have weaker effects and/or their protective effects may depend on other EMs. Thus, the analyses of single EMs may underestimate the effects of EMs on gut microbiota, and the mixture analyses are warranted to disentangle joint effects of EMs on gut microbiota in older adults. In this study, we aimed to examine the associations of single EMs and EM mixtures with the compositions of the gut microbiota using a sample of older adults in China.

## 2. Material and Methods

### 2.1. Data Source and Population

This study utilized data and biological specimens from the baseline survey of a cohort study: *Older Adult Health and Modifiable Factors*, which was launched in Fuyang city, Anhui province, China, from July to September 2018. Details on subject recruitment and sampling have been described elsewhere [22]. Briefly, a total of 6000 older adults aged 60 or over were randomly selected from 8 counties in Fuyang, and 5186 older adults agreed to participate in this survey. Each participant was invited to finish a structured questionnaire and undergo a physical examination in the local community hospital. Morning urine and stool samples were obtained from older adults when they underwent the physical examination. Only 300 fecal samples were collected due to the lack of refrigeration equipment at the investigation sites. Of 300 older adults with fecal samples, 30 were excluded because of insufficient feces (*n* = 17) or no urine sample (*n* = 13). Finally, a total of 270 older adults were included in the analysis. The protocol for this study was approved by the biomedical ethical committee of Anhui Medical University (No. 20190288), and all participants have provided written informed consent.

### 2.2. Measurement of Urinary EMs

The urine samples were removed from −80 °C to room temperature and melted. Then they were diluted to 10 times after mixed with diluent (0.05%TritonX-100 + 1%HNO_3_, Sigma, St. Louis, MO, USA) thoroughly. The Inductively Coupled Plasma Mass Spectrometer (ICP-MS; Nexion350X, Perkin-Elmer, Shelton, CT, USA) was used to measure urinary concentrations of seven EMs (including V, Co, Se, Sr, Mg, Ca, and Mo). The limits of detection (LODs) for V, Co, Se, Sr, Mg, Ca, and Mo were 0.005 µg/L, 0.001 µg/L, 0.274 µg/L, 0.015 µg/L, 0.002 mg/L, 0.0127 mg/L, and 0.002 µg/L, respectively. Urinary EM concentrations were corrected for urine dilution by urinary creatinine concentrations and expressed as µg/g creatinine. The urine creatinine concentrations were measured by using the picric acid assay.

### 2.3. 16S rRNA Gene Sequencing and Data Analyses

The 16S rRNA gene sequencing process was carried out in an entirely sterile environment. MagPure Stool DNA KF kit B was used to extract bacterial DNA in accordance with the manufacturer’s instructions (Magen, Guangzhou, China). The V4 hypervariable region of the 16S rRNA was amplified with degenerate PCR primers, 515F (5′-GTGCCAGCMGCCGCGGTAA-3′) and 806R (5′-GGACTACHVGGGTWTCTAAT-3′). The Illumina adapter, pad, and linker sequences were attached to both the forward and reverse primers. With the use of Agencourt AMPure XP beads and elution buffer, the PCR products were purified. Agilent Technologies’ 2100 bioanalyzer was used to qualify libraries. The validated libraries were used to generate 2 × 250 bp paired-end reads on the Illumina HiSeq 2500 platform (BGI, Shenzhen, China) using the standard Illumina pipelines.

The Fast Length Adjustment of Short Reads software (FLASH, v1.2.11) [23] was used to add paired-end reads to tags after raw reads had been filtered to eliminate adaptors and low-quality and ambiguous bases. The tags were grouped into operational taxonomic units (OTUs) with a cutoff value of 97%, and chimera sequences were compared with the Gold database using UCHIME (v4.2.40) [24]. Then, using the Ribosomal Database Project (RDP) Classifier v.2.2 and QIIME v1.8.0 with training data from the Greengenes database v201305, sample OTU sequences were taxonomically categorized [25]. The OTU abundance statistics table for each sample was obtained by comparing all Tags back to OTU using the USEARCH global [26].

### 2.4. Outcome Assessments

Based on the previous study [27], gut microbial alpha diversity, beta diversity, and the top 5 taxa in abundance at five levels from phylum to genus were reported, respectively. The Shannon index and inverse-Simpson were used to measure the richness and diversity of special taxon, respectively, which could be combined to comprehensively interpret the alpha diversity of gut microbiota. Beta diversity was measured by the Euclidean distance, and *R*^2^ of the analysis result was utilized to interpret the differences between samples.

### 2.5. Covariates

Covariates included demographic characteristics (age, gender, residence, education, economic condition, and body mass index (BMI)), behavioral factors (smoking, drinking, and antibiotic use), chronic diseases (hypertension, diabetes, and chronic kidney disease), and diet patterns.

Residence was dichotomized into rural or urban areas. Education was classified as illiteracy (without formal education), primary school (1–6 years of education) and junior school or above (>6 years of education). Economic condition was grouped into 2 categories (low and high) based on the self-perception. Behavioral factors were defined as follows: smoking status (non-smoker, former smoker, and current smoker), drinking status (never, often, and always), and antibiotic use (yes or no). 

In the process of physical examination, all participants were queried about their medical history and recent drug use. Medical histories included hypertension (yes or no), diabetes (yes or no), and chronic kidney disease (yes or no). We have double-checked the clinical history sheets, laboratory data, and other medical reports of each participant for ensuring the accuracy of the data. Additionally, diet consumption was measured by Food Frequency Questionnaire. All participants were asked whether they had eaten pork, vegetables, fruits, fungi, eggs, milk, and coarse cereals et al. in the past 12 months. If the respondents’ answer was “no”, the consumption frequency of this kind of food was recorded as “0”. If the respondents’ answer was “yes”, the consumption frequency was recorded according to how many times they eat every day/week/month/year. Diet consumption was clustered into 5 diet patterns based on factor analysis: factor 1 (mainly included Livestock meat, Fish meat, and Poultry), factor 2 (mainly included Soya, Animal viscera, Coarse cereals), factor 3 (mainly included Eggs, Milk, Nut, Sugary drinks, Fruits), factor 4 (mainly included Fungus, Pork), and factor 5 (mainly included Fruits, Animal oil, Vegetables), respectively. Details are provided in Supplementary Materials.

### 2.6. Statistical Analysis

The categorical variables, continuous variables, and the factor scores of diet patterns were described using frequencies and proportions, mean and standard deviation (SD), as well as range and median, respectively. The correlations between EMs, which had been adjusted by creatinine and log-transformed, were performed by Pearson correlation analysis.

We performed zero-inflated probabilistic principal component analysis (ZIPPCA) [28] using the mbDenoise R package to denoise substantial noise in microbiome data. More specifically, microbial count matrices contained a large proportion of zero values, and parts of them were caused by the low sequencing depth and sampling variations (technical zeros). The ZIPPCA denoises microbiome data by learning the latent features, which effectively deals with the data sparsity problem, distinguishes between technical and biological zeros, and then recovers the true abundance levels using the posterior mean. We then analyzed the alpha diversity (Shannon and inverse-Simpson indices), beta diversity (based on Euclidean distance), and the abundance of the taxon (the denoised counts of microbiome data).

Single- and multiple-element linear regression models were performed to examine the associations between single EMs and alpha diversity. Single-element linear models only included single EMs with and without adjusting covariates (age, gender, BMI, residence, education, economic condition, smoking, alcohol consumption, antibiotic use, medical history, and diet patterns). Multiple-element linear regression model included aforementioned covariates and all EMs. 

Furthermore, to assess the associations between single EMs and the beta diversity of gut microbiota, we divided each EM into two groups (high- and low-level groups) based on its median, and then compared the differences in beta diversity of gut microbiota between the two groups using the permutational analysis of variance (PERMANOVA) after adjustment for other EMs and aforementioned covariates. To more accurately estimate the relationship between each EM and the abundance of special taxon, we added pseudo counts of 1 to denoised counts of microbiome data before log transformation. The abundances of special taxon were then calculated and used as the dependent variables in subsequent multiple linear regression models. Independent variables and covariates were the same as the adjusted multiple linear regression models of alpha diversity.

Lastly, to examine joint associations of EMs with microbial metrics, we utilized Bayesian kernel machine regression (BKMR) [29] to flexibly model the associations of EMs with alpha diversity and the top five most abundant taxa in five levels from phylum to genus. Before fitting the BKMR models, the EMs concentrations were subtracted by the mean and then divided by the standard deviation. Given the high correlations between EMs, we divided EMs into two groups based on principal component analysis, and used a hierarchical variable selection approach to estimate the posterior inclusion probabilities (PIPs) for two groups as well as conditional PIPs (condPIPs) for each EM within the group, with the condPIPs > 0.5 indicating that the corresponding EMs were significant contributors to the variability of the outcomes. A *p* value < 0.05 was considered statistically significant in the current study, and all analyses were conducted in SPSS 26.0 and R 4.2.0.

## 3. Results

### 3.1. Population Characteristics

The study population consisted of 270 older adults with a mean age of 71.42 years old (SD = 4.91) and a mean BMI of 24.68 kg/m^2^ (SD = 3.74). Of 270 older adults, 51.9% were males, 75.2% resided in rural areas, 75.5% had lower education (≤primary school), 71.1% had never been drinking, 78.5% had never been smoking, and 70.0% had not used antibiotics in the past month. The factor scores of five diet patterns ranged from −8.173 to 10.792 (Table 1).

### 3.2. Gut Microbiota Compositions

**Figure 1:**
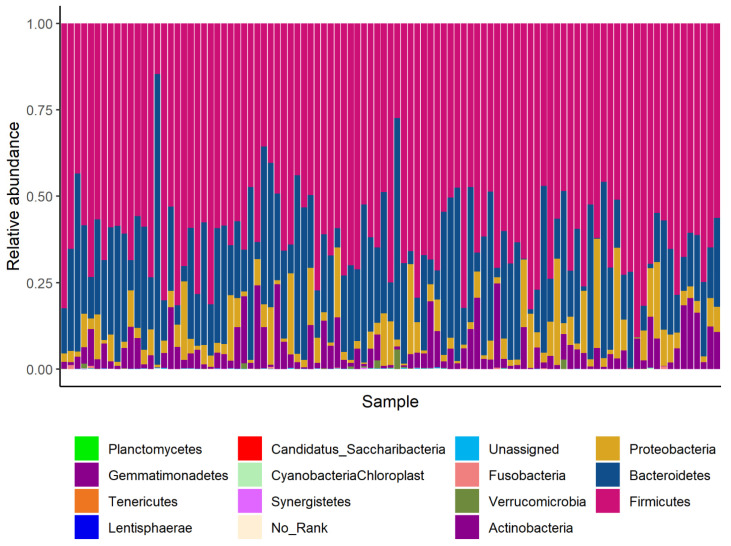
The denoised relative abundance of taxon at the phylum level.

A total of 1416 distinct OTUs were observed in the raw data. After excluding the OTUs that total counts were zero, the OTUs were assigned to 16 phyla, 28 classes, 51 orders, 84 families, and 178 genera, respectively. The denoised models were subsequently performed at different levels. Figure 1 shows the denoised relative taxon abundance at the phylum level. *Firmicutes* had the highest relative abundance in all bacterial phylum, followed by *Bacteroidetes* and *Proteobacteria*.

### 3.3. Distributions of Urinary EM Concentrations

Seven EMs, including V, Co, Se, Sr, Mg, Ca, Mo, were detected in all urine samples, and the corresponding median concentrations were 1.804 μg/L, 0.369 μg/L, 16.795 μg/L, 215.848 μg/L, 121.837 mg/L, 149.939 mg/L, and 116.160 μg/L, receptively (Table 2). The Pearson correlation coefficients between EMs are shown in Figure S1. There were significant positive correlations between all EMs, in which the associations between Sr, Mg, and Ca were accentuated, ranging from 0.56 to 0.85 (all *p* value < 0.001).

### 3.4. Associations of Single EMs with α-Diversity and β-Diversity

**Figure 2:**
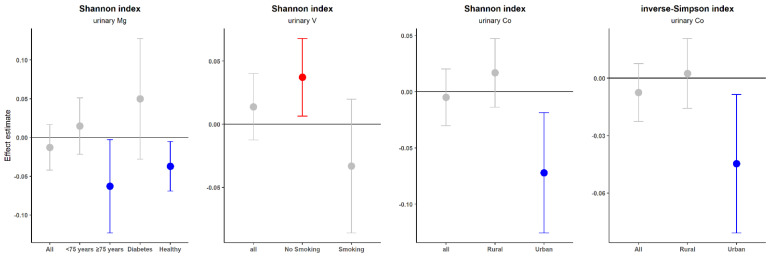
Associations of urine EMs with the Shannon and inverse−Simpson indices in the total sample and stratified by relevant covariates. Models were adjusted by age, gender, BMI, family income, region, smoking, drinking, physical activities, antibiotic use, diabetes, chronic kidney disease, hypertension, and the scores of five diet patterns.

The single-element and multiple-element linear regression models (Table S2) did not exhibit any statistically significant association between single EMs and α-diversity metrices (Shannon and inverse-Simpson indices) in total sample. BKMR models also showed that no EM significantly contributed to any metric of α-diversity (all condPIPs < 0.5; Table S6, Figure S2). However, stratified analyses found several significant associations in the subgroup population (Figure 2, Tables S3 and S4). Urinary V in non-smokers [*β* = 0.037,95%*CI* = (0.006, 0.068)], Co in urban older adults [*β* = −0.072, 95%*CI* = (−0.126, −0.019)], Mg in older adults age 75 years and above [*β* = −0.066, 95%*CI* = (−0.123, −0.003)], and Mg in those without diabetes [*β* = −0.037, 95%*CI* = (−0.069, −0.005) exhibited significant associations with the Shannon index. while urinary Co in urban older adults [*β* = −0.045, 95%*CI* = (−0.081, −0.008)] was also associated with the inverse-Simpson index. Similarly, no significant association between single EMs and *β*-diversity was found in the total sample, whereas significant differences in *β*-diversity were found between high- and low- level groups of urinary Sr levels (*R*^2^ = 0.023) and Ca levels (*R*^2^ = 0.035) in females, and of V levels (*R*^2^ = 0.095) in older adults who often drank (Table S5).

### 3.5. Associations between Single EMs and Specific Taxons 

In multivariable-adjusted regression models (Table S7), at least one element was detected in an association with the selected taxon in different levels except class level. The strongest negative association was detected between Mo and *Tenericutes* at the phylum level [*β* = −1.115, 95%*CI* = (−1.751, −0.479)], followed by the associations of Mo with *RF39* at the order level [*β* = −0.598, 95%*CI* = (−1.123, −0.073)] and Sr with *Bacteroides* at the order level [*β* = −0.408, 95%*CI* = (−0.754, −0.061)]. The strongest positive association was found between Mo and *Megamonas* at the genus level [*β* = 0.681, 95%*CI* = (0.248, 1.113)], followed by the associations of Sr with *Bifidobacteriales* at the order level [*β* = 0.412, 95%*CI* = (0.150, 0.673)], Mo with *Bacteroides* at the genus level [*β* = 0.385, 95%*CI* = (0.066, 0.703)], and Ca with *Bacteroidales* at the order level [*β* = 0.373, 95%*CI* = (0.078, 0.668)]. Additionally, although no association between the selected taxon whose abundances ranked as the top 5 at class level and EMs was significant, the *p* values for the associations between Mg and each taxon were all smaller than 0.07. 

**Figure 3:**
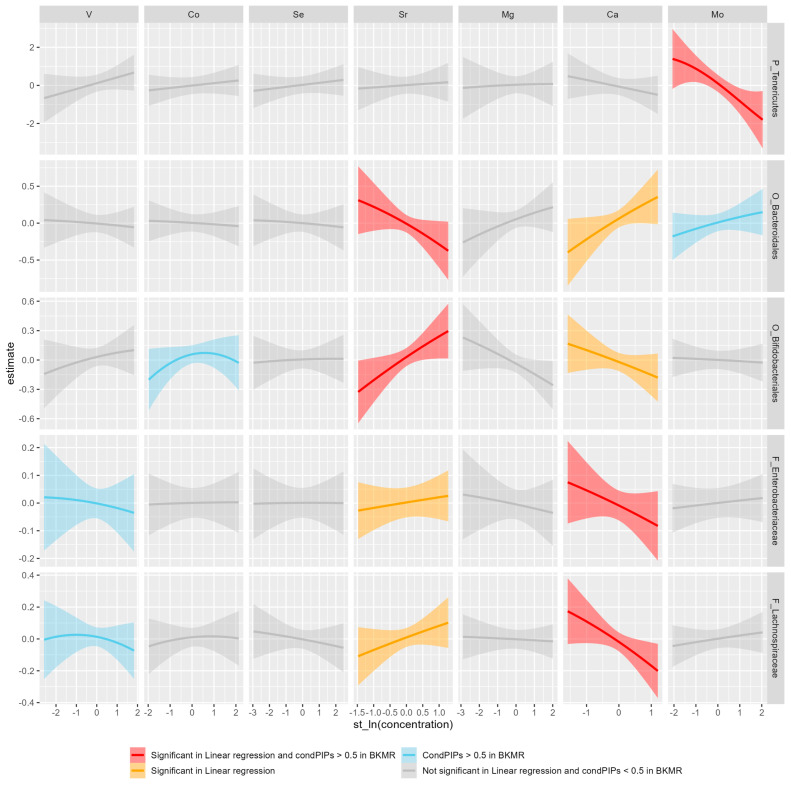
Univariate exposure response functions and 95% confidence intervals (*CIs*) for single EMs when all other exposures are fixed at the median, for taxa whose anyone EMs’ CondPIP > 0.5 in BKMR models. The color indicates the significance in linear regressions or BKMR models. All models were adjusted by age, gender, BMI, family income, region, smoking, drinking, physical activities, antibiotic use, diabetes, chronic kidney disease, hypertension, and the scores of five diet patterns.

We next used BKMR to replicate the results identified in multiple regression models. The CondPIPs for significant EMs that were identified using BKMR models ranged from 0.110 for Sr contributing to the abundance of *Enterobacteriaceae* at family level to 0.975 for Sr contributing to the abundance of *Bacteroides* at genus level (Table S8). Of these EMs, Sr (in an association with *Bacteroides* at the genus level and with *Bifidobacteriales* and *Bacteroidales* at the order level), Mo (with *Tenericutes* at the phylum level), and Ca (with *Lachnospiraceae* and *Enterobacteriaceae* at the family level) had condPIP values of >0.5 and exhibited the same association direction as those described in multiple regression models (Figure 3 and Figure S3–S7). Furthermore, dose–response curves from BKMR suggested a nonlinear (inverted U-shaped) association between Sr and *Bacteroides* at the genus level.

### 3.6. The Cumulative Effects of EMs on the Compositions of the Gut Microbiota 

**Figure 4:**
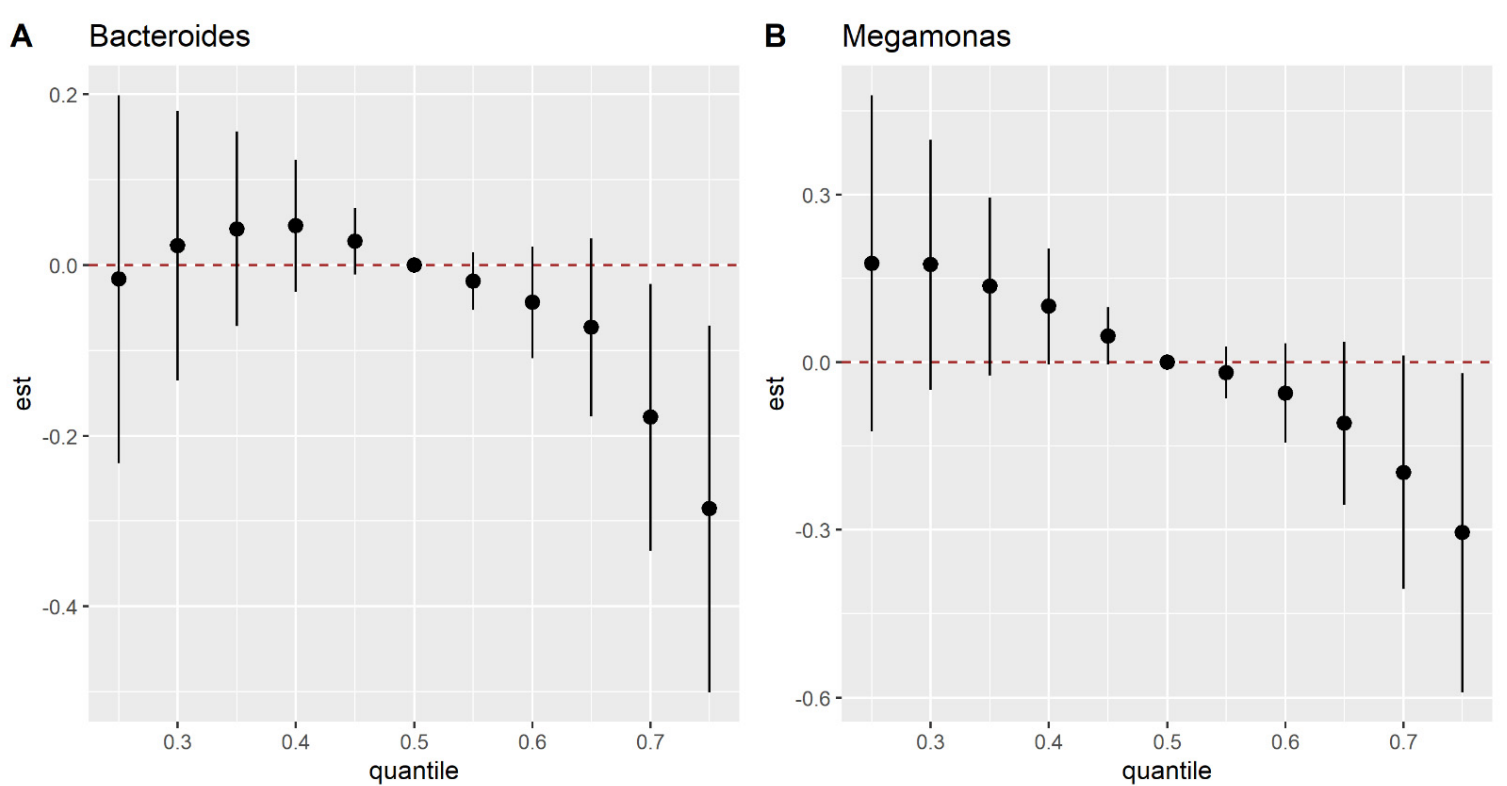
Joint exposure effect of the mixture (95% *CIs*) for *Bacteroides* (**A**) and *Megamonas* (**B**), defined as the predicted change in the outcomes when all EMs were held at particular percentiles, compared to when all metals were held at their median concentrations.

The overall effect of the EM mixture on α-diversity is shown in Figure S8. There was a linear increase in the Shannon index or inverse-Simpson index with the elevated levels of the mixture, although no statistical significance was found. The overall effect of the EM mixture on specific taxon in different levels exhibited complex pictures (Figure S9). Of these associations, the EM mixture exhibited significantly negative associations with the abundance of *Bacteroides* and *Megamonas* at the genus level when all EMs were fixed at the 80th or above percentile (Figure 4).

### 3.7. The Interaction Effects of EMs on the Composition of the Gut Microbiota

The bivariate exposure response functions, provided by BMKR, were used to identify the potential interactions. Although there was no obvious interaction between EMs on α-diversity (Figures S10 and S11) and the above-mentioned five significant taxons (Figures S12–S16), we found there were interactions between Sr and Mo in their associations with *Bacteroides* and between V and Mo in their associations with *Megamonas* (Figures S17 and S18).

**Figure 5:**
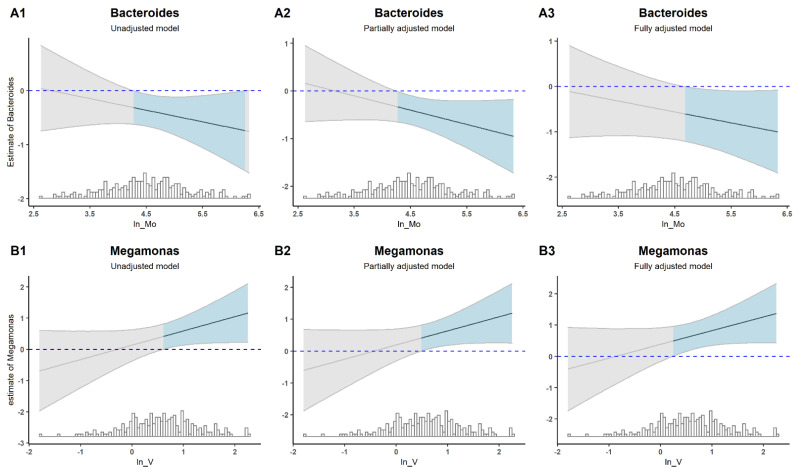
Regression estimates of Sr effects (the line in shaded areas) and 95% confidence interval (shaded areas) on *Bacteroides* as a function of Mo (**A1**–**A3**); Mo effects (the line in shaded areas) and 95% confidence interval (shaded areas) on *Megamonas* as a function of V (**B1**–**B3**). Unadjusted models only included the two EMs and their interaction items; Partially adjusted models adjusted covariates on the basis of the unadjusted models; Fully adjusted models adjusted other EMs on the basis of the partially adjusted models.

We then applied multi-variable linear regression models to confirm these interaction effects. Given the limited power of this model, the *p* value of interaction terms (*P_int_*) < 0.3 could be considered as significant. We found that the effect of interaction between Sr and Mo on *Bacteroides* was significant when the Mo exposure was relatively high (*P_int_* = 0.132–0.274), and the direction of the interaction suggested that Sr and Mo had a negative synergistic relationship (Figure 5A1–A3). The *p* values of the interaction term of V and Mo on *Megamonas* ranged from 0.082 to 0.098. Those models all showed significant interaction effects of V and Mo on the abundance of *Megamonas* when the V exposure was relatively high and with a positive synergistic relationship between them (Figure 5B1–B3).

## 4. Discussion

The main findings of our study were as follows: (1) no single EMs were associated with the altercations in the gut microbiota diversity and community structure in the total sample, individually, and as a mixture, whereas several significant associations of single EMs (V, Co, Mg, Sr, and Ca) with α-diversity and/or *β*-diversity were found in subgroups of older adults; (2) both multiple linear regression and BKMR showed that Sr, Mo, and Ca significantly contributed to the abundance of several bacterial taxons at different levels: Sr (in an association with *Bacteroides* at the genus level and with *Bifidobacteriales* and *Bacteroidales* at the order level), Mo (with *Tenericutes* at the phylum level), and Ca (with *Lachnospiraceae* and *Enterobacteriaceae* at the family level); (3) the EM mixture exhibited a linear dose–response association with the Shannon or inverse-Simpson indices, although no significance was found; (4) the EM mixture showed significantly negative associations with the abundance of *Bacteroides* and *Megamonas* at the genus level, in which Sr and Mo had an interaction on *Bacteroides*, and V and Mo had an interaction on *Megamonas*. To our best knowledge, this is the first study to explore the associations between multiple EMs and gut microbiota in older adults. Our study suggested that EMs may play an important role in maintaining the steady status of gut microbiota.

Urine samples have been widely used to assess individual exposure to EMs. The median urine concentrations of the EMs in our sample (μg/L) were comparable to those found in other research. For instance, the urine Co concentration of 0.369 μg/L was marginally lower than the reported value of Xiangdong Wang (0.389 μg/L) [30], while the urine V concentration of 1.804 μg/L was marginally higher than the value reported by Shunli Jiang (1.27 μg/L) [31]. These differences could result from the specific population, diet, lifestyle, and so on. Given that diets, lifestyles, and daily activities in older adults are more stable than those of other populations, such as teenagers, we believe that the urine EMs concentrations based on the cross-sectional investigation may be used to estimate or replace the exposure levels in the past period.

Although both single- and multiple-element linear regression models did not find a significant association between Sr and alpha diversity, the BKMR models exhibited that Sr was the most important contributor within the EM mixture to alpha diversity, which was similar to the report from a prospective cohort study of Chinese pregnant women [32]. Furthermore, Sr showed a negative association with *Bacteroidales* (at the order and genus levels) and a positive association with *Bifidobacteriales* (at the order level). So far, no comparable study has been found. It is well known that Sr ions (${\mathrm{Sr}}^{2+}),$ resembling Ca ions (${\mathrm{Ca}}^{2+})$, are bound to phosphate in the bones and have the potential for preventing osteoporosis. A recent study [33] found that the order *Bacteroidales* and family *Lachnospiraceae* were negatively associated with bone mass, which, together with our findings, suggests a possible link between Sr and *Bacteroidales.* Whether *Bacteroidales* mediates the effect of the Sr on bone health is warranted to be investigated.

Excessive Mo intake could lead to diarrhea, which suggested that Mo may interfere with gut microbial metabolism. A recent animal study using a 2 × 2 factorial design to examine the effect of diet with Mo on the gut microbiota in laying hens found that high Mo levels in the diet led to lower *Firmicutes* and higher *Proteobacteria* abundance, possibly disrupting redox balance and reducing production performance [34]. However, high Mo level in experimental animals is unlikely to occur in the human body. The relationship between Mo and gut microbiota in the human body remains unknown. In this study, Mo was not associated with gut microbiota diversity as a whole, whereas it exhibited a negative association with *Tenericutes* and positive associations with *Bacteroides* and *Megamonas* in both the linear and BKMR models. *Tenericute*, *Bacteroides*, and *Megamonas* are all the dominant bacteria in the gut, and the latter two could produce butyric acid. Our findings suggested that moderate Mo supplements in older adults may help the growth of beneficial bacteria in the gut. 

Animal studies have reported increased alpha diversity in those rats or mice fed with a high Ca diet [17,35]. The possible mechanism by which Ca beneficially modifies the gut microbiota is via precipitating bile acids and fatty acids and reducing cytotoxicity to the intestinal mucosa [36,37]. However, human studies on the association of Ca with gut microbiota did not yield consistent findings. For instance, Falak Zeb et al. [38] conducted a randomized controlled trial and reported that there was a negative association between Ca and alpha diversity. Similarly, Lara S. Yoon et al. [21] performed a randomized crossover design where three groups of study participants with a supplement of calcium alone, inulin alone, or both calcium and inulin did not exhibit significant differences in alpha diversity and composition of gut microbiota. The reasons for the mixed results remain unclear. In addition to sample sizes, supplemental dose, and intervention duration, another possibility is that the effect of Ca on gut microbiota is dependent on other EMs. In this study, both single-element and mixture models were used to examine the association between Ca and gut microbiota. We observed that Ca was positively associated with the Shannon and inverse-Simpson indices, individually and as a mixture, although no significance was found. Moreover, a small but significant difference was found in *β*-diversity between high and low levels of urine Ca exposure in females.

*Firmicutes* and *Bacteroidetes* were the dominant bacteria of the human gut microbiota, and the *Firmicutes*/*Bacteroidetes* (F/B) ratio was related to obesity in recent years [39,40]. In our study, we found that urinary Ca had a negative association with the F/B ratio, which aligns with an animal study [16] in which dietary calcium promoted a significant increase in *Bacteroidetes* and *Actinobacteria*. Additionally, there was a negative and significant association between urinary Ca and the abundance of *Lachnospiraceae* in our results, which was similar to reports by Li et al. [41], where the abundance of *Lachnospiraceae* significantly decreased in the mice fed normal Ca as compared with those fed with low-level Ca. A previous study indicated that dietary calcium intake could increase the intraluminal calcium concentration to stimulate gastrin release and acid secretion, and further prohibit the growth of acid-intolerant bacteria, such as *Lachnospiraceae* [36]. 

A major strength of our study is that we utilized a new denoised method to deal with the count data of gut microbiota, which effectively avoided the troublesome zero-inflated nature of microbiome data and ensured the quality of downstream analysis. Secondly, applying the BKMR model helped us to identify which EMs provided the greatest contribution to the outcomes, and the revealed non-linear association and interaction may be more meaningful for future validation studies. Likewise, the limitations of this study also need to be acknowledged and discussed. First, food consumption frequencies rather than consumption quantities were obtained, which may not be accurate measurements for dietary intakes and could lead to residual confounding. Second, the cross-sectional nature of this study restricted causal inferences. Finally, the 16S rRNA amplicon sequencing cannot identify the bacteria at the genus and the species level, which may result in erroneous conclusions.

## 5. Conclusions

In summary, our study found some novel associations of common essential nutrient elements with the gut microbiota diversity and specific taxons with a higher abundance in older adults: The negative and linear associations of Mo with *Tenericutes*, Sr with *Bacteroidales*, and Ca with *Enterobacteriaceae* and *Lachnospiraceae*, and a positive and linear association of Sr with *Bifidobacteriales* were found. Our findings suggested that EMs may play an important role in maintaining the steady status of gut microbiota. Future prospective epidemiological studies with metagenomic sequencing are needed to replicate these findings, as well as to further elucidate the additional contribution of the EMs to the gut microbiota.

## Figures and Tables

**Table 1:** Distribution of demographics and potential covariates.

Basic Characteristics	Levels	*n* (%)
Categorical variable		
Gender	Male	140(51.9)
	Female	130(48.1)
Education	Illiteracy	128(47.4)
	Primary school	76(28.1)
	Junior school or above	66(24.4)
Family income	Low income	200(74.1)
	High income	70(25.9)
Region	Rural	203(75.2)
	Urban	67(24.8)
Smoking	Yes	58(21.5)
	No	212(78.5)
Drinking	Never	192(71.1)
	Often	36(13.3)
	Always	42(15.6)
Physical activities	Low level	95(35.2)
	Moderate level	78(28.9)
	Severe level	97(35.9)
Antibiotic used	Yes	81(30.0)
	No	189(70.0)
Diabetes	Yes	92(34.1)
	No	178(65.9)
CKD	Yes	37(13.7)
	No	233(86.3)
Hypertension	Yes	171(63.3)
	No	99(36.7)
Continuous variables		Mean ± SD/Range(median)
Age (years)	-	71.422 ± 4.911
BMI (kg/m^2^)	-	24.675 ± 3.739
Dietary pattern1	-	−2.703–10.792(−0.262)
Dietary pattern2	-	−1.382–9.073(−0.255)
Dietary pattern3	-	−2.005–4.694(−0.227)
Dietary pattern4	-	−1.517–6.367(−0.282)
Dietary pattern5	-	−8.173–2.648(0.043)

Note: SD, standard deviation; CKD, Chronic kidney disease.

**Table 2:** Detection rates and urinary concentrations of EMs in study population (*n* = 270).

Elements	LOD	DR%	GM	Unadjusted (Creatinine Adjusted) ^a^				
				5th	25th	50th	75th	95th
V	0.005	100%	1.804 (2.225)	0.746 (0.532)	1.238 (1.088)	1.730 (1.773)	2.258 (2.720)	3.150 (5.673)
Co	0.001	100%	0.369 (0.405)	0.010 (0.117)	0.182 (0.201)	0.289 (0.300)	0.443 (0.462)	0.941 (1.238)
Se	0.274	100%	16.795 (17.374)	3.892 (4.678)	8.855 (10.657)	13.867 (15.179)	20.518 (20.641)	37.616 (38.424)
Sr	0.015	100%	215.848 (233.08)	51.942 (56.235)	100.366 (112.627)	178.797 (192.590)	282.117 (331.390)	540.303 (557.702)
Mg	0.0022	100%	121.837 (124.291)	21.142 (34.060)	60.933 (69.872)	103.073 (105.487)	149.911 (150.942)	293.133 (321.088)
Ca	0.0127	100%	149.939 (159.150)	23.140 (30.732)	59.810 (63.997)	113.075 (128.613)	202.794 (225.834)	391.535 (405.582)
Mo	0.002	100%	116.160 (115.540)	18.992 (24.983)	44.287 (56.391)	87.116 (89.089)	159.657 (138.747)	340.495 (315.389)

Note: ^a^ Unadjusted: volume-based urinary EMs concentrations (μg/L: V, Co, Se, Sr, Mo; mg/L: Mg, Ca); Creatinine adjusted: urinary EMs concentrations were corrected for urine dilution by urinary creatinine levels (μg/g creatinine). LOD, limits of detection (μg/L: V, Co, Se, Sr, Mo; mg/L: Mg, Ca); DR, detection rate; GM, Geometric mean.

## Data Availability

Not applicable.

## Supplementary Materials

The following supporting information can be downloaded at: https://www.mdpi.com/article/10.3390/nu15051137/s1. Figure S1: Pearson correlation of ln creatinine-adjusted urinary EMs concentration; Figure S2: Univariate exposure response functions and 95% confidence intervals (*CIs*) for single EMs when all other exposures are fixed at the median. Figure S3: Univariate exposure response functions and 95% confidence intervals (*CIs*) for single EMs when all other exposures are fixed at the median, for the top 5 most abundant taxa at the phylum level. Figure S4: Univariate exposure response functions and 95% confidence intervals (*CIs*) for single EMs when all other exposures are fixed at the median, for the top 5 most abundant taxa at the class level. Figure S5: Univariate exposure response functions and 95% confidence intervals (*CIs*) for single EMs when all other exposures are fixed at the median, for the top 5 most abundant taxa at the order level. Figure S6: Univariate exposure response functions and 95% confidence intervals (*CIs*) for single EMs when all other exposures are fixed at the median, for the top 5 most abundant taxa at the family level. Figure S7: Univariate exposure response functions and 95% confidence intervals (*CIs*) for single EMs when all other exposures are fixed at the median, for the top 5 most abundant taxa at the genus level. Figure S8: Joint exposure effect of the mixture (95% *CIs*) for Shannon index (A) and Inverse-Simpson index (B). Figure S9: Joint exposure effect of the mixture (95% *CIs*) for all selected taxa. Figure S10: Bivariate exposure response functions for Shannon index. Figure S11: Bivariate exposure response functions for Inverse-Simpson index. Figure S12: Bivariate exposure response functions for *Tenericutes*. Figure S13: Bivariate exposure response functions for *Bacteroidales*. Figure S14: Bivariate exposure response functions for *Bifidobacteriales*. Figure S15: Bivariate exposure response functions for *Enterobacteriaceae*. Figure S16: Bivariate exposure response functions for *Lachnospiraceae*. Figure S17: Bivariate exposure response functions for *Bacteroides*. Figure S18: Bivariate exposure response functions for *Megamonas*. Table S1: Factor loadings of five dietary patterns ^a^; Table S2: Associations between urinary concentrations of EMs and the α-diversity; Table S3: Association between urinary concentrations of EMs and Shannon index for multiple-elements models. Table S4: Association between urinary concentrations of EMs and inverse-Simpson index for multiple-elements models. Table S5: Association of urinary EMs concentration with β-diversity (Euclidean distance). Table S6: Posterior Inclusion Probabilities (PIPs) for EMs associated with α-diversity metrics. Table S7: Associations of ln creatinine-adjusted urinary EMs concentration with log denoised counts of individual bacterial taxa. Table S8: The condPIPs for EMs associated with log denoised counts of individual bacterial taxa.

## Abbreviations

BKMR: Bayesian Kernel Machine Regression; BMI, body mass index; Ca, calcium; CI, confidence interval; Co, cobalt; CondPIPs, conditional posterior inclusion probabilities; EMs, essential metal(loid)s; LOD, limit of detection; Mg, magnesium; Mo, molybdenum; OUT, operational taxonomic unit; SD, standard deviation; Se, selenium; Sr, strontium; V, vanadium; ZIPPCA, zero-inflated probabilistic principal components analysis.
