# Supplementary material for: The Associations between Multiple Essential Metal(loid)s and Gut Microbiota in Chinese Community-Dwelling Older Adults
Source: Nutrients. 2023 Feb 24;15(5):1137. doi: 10.3390/nu15051137 (PMC10005492; doi:10.3390/nu15051137)
Supplement: Supplementary file 1 [file nutrients-15-01137-s001.zip › nutrients-2120421-supplementary.pdf]

Supplemental materials

The factor analysis of dietary patterns

We investigated the frequency of 15 common foods, including pork, poultry, livestock meat, fish meat, vegetables, fruits, sugary drinks, nut, soya, fungus, eggs, animal viscera, milk, animal oil and coarse cereals, and performed a factor analysis to identify dietary patterns. The value of KMO was 0.658, and the *P*-value of Bartlett's test was <0.001. The result of analysis showed that, according to the eigenvalue roots >1, all foods were clustered into 5 dietary categories, see Table 1S, and the cumulative variance contribution rate of five dietary pattens was 52.243%, in turn, 12.110%, 11.387%, 10.984%, 9.642%, 8.120%. The calculated each factor score would be as the covariates included in the subsequently analyses.

Table S1 Factor loadings of five dietary patterns[a]

Factor 1		Factor 2		Factor3		Factor4		Factor5	
Food	Factor loadings	Food	Factor loadings	Food	Factor loadings	Food	Factor loadings	Food	Factor loadings
Livestock meat	0.777	Soya	0.819	Eggs	0.667	Fungus	0.815	Fruits	0.379
Fish meat	0.731	Animal viscera	0.794	Milk	0.640	Pork	0.771	Animal oil	-0.690
Poultry	0.717	Coarse cereals	0.584	Nut	0.548			Vegetables	0.595
				Sugary drinks	0.413				
				Fruits	0.398				

a indicates only display the food with factor loadings >0.3.

Table S2 Associations between urinary concentrations of EMs and the α-diversity

Alpha diversity index	Elements	Single-element(unadjusted)		Single-element(adjusted)		Multiple-elements (adjusted)	
		β（95%CI）	*P*	*β*（95%CI）	*P*	*β*（95%CI）	*P*
Shannon	V	0.014(-0.006,0.035)	0.175	0.014(-0.007,0.035)	0.197	0.014(-0.013,0.040)	0.305
	Co	0.006(-0.015,0.027)	0.544	0.003(-0.018,0.024)	0.780	-0.005(-0.030,0.020)	0.699
	Se	0.003(-0.020,0.026)	0.799	0.009(-0.014,0.032)	0.436	0.004(-0.024,0.031)	0.803
	Sr	0.003(-0.018,0.024)	0.783	0.007(-0.014,0.028)	0.518	-0.017(-0.061,0.026)	0.435
	Mg	<0.001(-0.022,0.022)	0.985	0.002(-0.021,0.024)	0.887	-0.013(-0.042,0.017)	0.399
	Ca	0.007(-0.011,0.025)	0.452	0.010(-0.008,0.028)	0.295	0.021(-0.015,0.058)	0.255
	Mo	0.016(-0.004,0.036)	0.110	0.010(-0.010,0.031)	0.322	0.011(-0.012,0.034)	0.328
inverse-Simpson	V	0.005(-0.007,0.017)	0.413	0.006(-0.006,0.019)	0.330	0.005(-0.011,0.021)	0.549
	Co	-0.001(-0.013,0.011)	0.875	-0.001(-0.014,0.011)	0.843	-0.008(-0.023,0.008)	0.332
	Se	0.001(-0.012,0.015)	0.851	0.005(-0.009,0.019)	0.477	0.001(-0.015,0.018)	0.865
	Sr	0.005(-0.007,0.017)	0.416	0.007(-0.005,0.020)	0.243	-0.004(-0.030,0.022)	0.759
	Mg	0.005(-0.008,0.018)	0.433	0.006(-0.008,0.019)	0.396	-0.002(-0.019,0.016)	0.864
	Ca	0.006(-0.004,0.016)	0.252	0.008(-0.002,0.019)	0.128	0.011(-0.011,0.033)	0.329
	Mo	0.008(-0.003,0.020)	0.157	0.006(-0.007,0.018)	0.358	0.005(-0.009,0.019)	0.469

Single- and Multiple-models were adjusted by age, gender, BMI, family income, region, smoking, drinking, physical activities, antibiotic use, diabetes, chronic kidney disease, hypertension, and the scores of five dietary patterns.

Table S3 Association between urinary concentrations of EMs and Shannon index for multiple-elements models

Covariates	Level	N	Vanadium	Cobalt	Selenium	Strontium	Magnesium	Calcium	Molybdenum
Not stratified	-	270	0.014	-0.005	0.004	-0.017	-0.013	0.021	0.011
Gender	Male	140	0.015	-0.020	0.007	-0.001	-0.027	0.021	0.009
	Female	130	0.015	0.015	-0.004	-0.046	-0.004	0.031	0.013
Age	<75 years	198	0.005	0.008	-0.010	-0.021	0.015	0.010	0.019
	≥75 years	72	0.021	0.003	0.019	-0.027	**-0.063**	0.048	-0.010
BMI	Low-weight or normal	150	-0.001	-0.012	0.006	-0.026	-0.016	0.048*	0.008
	Overweight or obese	120	0.015	0.010	-0.003	-0.029	-0.024	0.005	0.022
Region	Rural	203	0.005	0.017	0.004	-0.032	-0.001	0.018	0.012
	Urban	67	0.006	**-0.072**	0.029	0.050	-0.037	0.012	0.040
Antibiotic use	Yes	81	-0.022	-0.012	0.053	-0.033	0.010	0.035	0.026
	No	189	0.018	0.001	-0.011	-0.009	-0.011	0.010	<0.001
Smoking	Yes	58	-0.033	0.030	-0.021	0.046	-0.036	-0.015	0.023
	No	212	**0.037**	-0.011	0.008	-0.049*	-0.006	0.034	0.022*
Drinking	Never	192	0.011	0.008	0.011	-0.031	-0.017	0.032	0.014
	Often	36	0.009	-0.057	-0.012	-0.093	-0.001	0.074	-0.052
	Always	42	0.056	-0.087	0.001	-0.080	-0.008	0.039	0.058
Physical activities	Low	95	0.015	0.022	-0.015	-0.019	0.011	0.013	0.022
	Moderate	78	0.019	-0.022	-0.014	-0.037	0.026	0.052	0.019
	Severe	97	0.010	-0.021	0.047*	0.008	-0.047	0.001	0.026
Hypertension	Healthy	99	-0.004	0.034	0.001	0.025	-0.004	-0.024	0.022
	Patient	171	0.022	-0.021	0.002	-0.019	-0.020	0.030	0.009
Diabetes	Healthy	178	-0.001	-0.003	0.012	0.015	**-0.037**	0.005	0.012
	Patient	92	0.027	0.029	-0.009	-0.050	0.050	0.003	-0.007

* indicates *P* value < 0.1, **bold** indicates *P* value < 0.05; Models were adjusted by age, gender, BMI, family income, region, smoking, drinking, physical activities, antibiotic use, diabetes, chronic kidney disease, hypertension, and the scores of five dietary patterns (except where stratified by that variable).

Table S4 Association between urinary concentrations of EMs and inverse-Simpson index for multiple-elements models

Covariates	Level	N	Vanadium	Cobalt	Selenium	Strontium	Magnesium	Calcium	Molybdenum
Not stratified	-	270	0.005	-0.008	0.001	-0.004	-0.002	0.011	0.005
Gender	Male	140	0.010	-0.016	0.004	0.009	-0.003	-0.005	0.002
	Female	130	-0.002	0.002	0.002	-0.024	-0.007	0.035*	0.006
Age	<75 years	198	0.004	-0.005	-0.012	<0.001	0.006	0.010	0.012
	≥75 years	72	0.006	0.001	0.020	-0.019	-0.010	0.004	-0.006
BMI	Low-weight or normal	150	-0.001	-0.013	0.004	-0.011	-0.006	0.027	0.006
	Overweight or obese	120	0.004	0.005	-0.004	-0.012	-0.007	0.002	0.010
Region	Rural	203	0.002	0.003	-0.001	-0.011	0.003	0.010	0.009
	Urban	67	0.07	**-0.045**	0.040*	0.012	0.011	0.008	-0.005
Antibiotic use	Yes	81	-0.001	-0.012	0.026	-0.028	-0.008	0.038	0.011
	No	189	0.004	-0.005	-0.005	0.002	0.002	0.002	<0.001
Smoking	Yes	58	-0.009	0.014	-0.017	0.036	-0.007	-0.018	<0.001
	No	212	0.011	-0.009	0.006	-0.022	0.002	0.019	0.012
Drinking	Never	192	0.001	<0.001	0.005	-0.008	-0.005	0.019	0.002
	Often	36	0.001	-0.026	-0.064	-0.012	0.024	0.024	-0.009
	Always	42	0.032	-0.062*	0.024	-0.042	-0.009	0.016	0.031
Physical activities	Low	95	-0.016	0.011	0.003	0.004	0.017	0.018	0.007
	Moderate	78	-0.005	-0.022	-0.008	0.003	0.013	0.017	0.018
	Severe	97	0.016	-0.011	0.019	-0.004	-0.019	-0.002	0.013
Hypertension	Healthy	99	-0.006	-0.013	-0.010	0.003	0.016	-0.001	0.012
	Patient	171	0.008	-0.019*	0.006	0.001	-0.008	0.011	0.003
Diabetes	Healthy	178	-0.001	-0.007	0.015	-0.002	-0.008	0.008	0.001
	Patient	92	0.008	0.005	-0.021	-0.001	0.014	0.006	0.012

* indicates *P* value< 0.1, bold indicates *P* value< 0.05; Models were adjusted by age, gender, BMI, family income, region, smoking, drinking, physical activities, antibiotic use, diabetes, chronic kidney disease, hypertension, and the scores of five dietary patterns (except where stratified by that variable).

Table S5 Association of urinary EMs concentration with β-diversity (Euclidean distance).

Covariates	Level	N	R^2						
			Vanadium	Cobalt	Selenium	Strontium	Magnesium	Calcium	Molybdenum
Not stratified	-	270	0.002	0.003	0.003	<0.001	0.002	0.002	0.003
Gender	Male	140	0.004	0.012	0.009	0.020*	0.007	0.011	0.009
	Female	130	0.003	0.002	0.006	**0.023**	0.010	**0.035**	0.008
Age	<75 years	198	0.004	0.007	0.008	0.006	0.003	0.006	0.002
	≥75 years	72	0.013	0.004	0.003	0.024	0.004	0.004	0.021
BMI	Low weight or normal	150	0.005	0.004	<0.001	<0.001	0.006	<0.001	0.012
	Overweight or obese	120	0.007	0.005	0.010	0.002	0.009	0.007	0.004
Region	Rural	203	0.003	0.003	0.001	<0.001	0.004	0.002	0.004
	Urban	67	0.003	0.012	0.011	0.008	0.004	0.010	0.017
Antibiotic use	Yes	81	0.026	0.007	0.014	0.011	0.003	0.002	0.012
	No	189	<0.001	0.006	0.002	0.001	0.004	0.002	0.003
Smoking	Yes	58	0.009	0.001	0.002	0.015	0.001	0.003	0.012
	No	212	0.007	0.004	0.006	0.001	0.003	0.003	0.010
Diabetes	Healthy	192	0.001	0.001	0.005	0.008	<0.001	0.001	0.002
	Patient	36	0.019	0.032*	0.005	0.013	0.012	0.021	0.010
Drinking	Never	42	<0.0001	0.002	0.001	0.003	0.002	0.010	0.002
	Often	95	**0.095**	0.037	0.017	0.008	0.002	0.014	0.038
	Always	78	0.001	0.002	0.033	0.037	0.011	0.035	0.056*
Physical activities	Low	97	0.002	0.008	<0.001	0.005	0.020	0.010	0.002
	Moderate	99	0.005	0.001	0.001	0.011	0.021	0.016	0.010
	Severe	171	0.006	0.028*	0.013	0.009	0.009	0.007	0.011
Hypertension	Healthy	178	0.008	0.006	0.026*	0.018	0.010	0.029*	0.004
	Patient	92	0.004	0.005	0.002	0.006	0.001	0.003	0.003
Diabetes	Healthy	270	0.001	0.001	0.005	0.008	<0.001	0.001	0.002
	Patient	140	0.019	0.032*	0.005	0.013	0.012	0.021	0.010

* indicates $P < 0.1$, **bold** indicates $P < 0.05$; Models were adjusted by age, gender, BMI, family income, region, smoking, drinking, physical activities, antibiotic use, diabetes, chronic kidney disease, hypertension, and the scores of five dietary patterns (except where stratified by that variable)

Table S6 Posterior Inclusion Probabilities (PIPs) for EMs associated with α-diversity metrics

Exposure	group	Shannon		inverse-Simpson	
		groupPIP	condPIP	groupPIP	condPIP
V	1	0.154	0.307	0.106	0.339
Co	1	0.154	0.132	0.106	0.158
Se	1	0.154	0.129	0.106	0.121
Sr	2	0.217	0.292	0.273	0.422
Mg	2	0.217	0.335	0.273	0.162
Ca	2	0.217	0.373	0.273	0.416
Mo	1	0.154	0.431	0.106	0.383

Table S7 Associations of ln creatinine-adjusted urinary EMs concentration with log denoised counts of individual bacterial taxa

Level	taxon	V	Co	Se	Sr	Mg	Ca	Mo
Phylum	*Firmicutes*	0.016	0.062	0.017	0.028	0.020	-0.099	-0.048
	Bacteroidetes	-0.122	-0.103	-0.017	-0.071	0.034	0.233	0.062
	Proteobacteria	0.2368	-0.063	-0.003	-0.009	-0.051	-0.108	-0.027
	Actinobacteria	-0.015	0.049	-0.024	0.215	-0.121	-0.191	0.085
	Tenericutes	0.559	0.282	0.424	0.468	0.187	-0.901*	**-1.115**
Class	*Clostridia*	-0.003	-0.013	-0.002	-0.023	0.031*	0.012	-0.002
	Bacteroidia	-0.008	-0.035	-0.004	-0.063	0.085*	0.033	-0.006
	Gammaproteobacteria	0.003	0.017	0.002	0.030	-0.040*	-0.016	0.003
	Bacilli	0.016	0.096	0.016	0.157	-0.213*	-0.080	0.017
	Actinobacteria	0.020	0.123	0.022	0.200	-0.271*	-0.101	0.022
Order	*Clostridiales*	0.028	0.047	-0.013	-0.027	0.040	0.026	-0.025
	Bacteroidales	-0.048	-0.030	-0.041	**-0.408**	0.137	**0.373**	0.138
	Enterobacteriales	0.017	-0.004	-0.011	0.046	-0.019	0.006	0.022
	RF39	0.422	0.424	0.195	-0.011	0.187	-0.338	**-0.598**
	Bifidobacteriales	0.079	0.016	0.008	**0.412**	-0.146	**-0.261**	-0.051
Family	*Ruminococcaceae*	0.041	0.084*	0.014	0.118	0.045	-0.105	**-0.116**
	Prevotellaceae	-0.020	-0.059	0.029	-0.150*	-0.029	**0.148**	0.061
	Enterobacteriaceae	-0.028	-0.006	-0.023	**0.197**	-0.043	**-0.175**	0.041
	Veillonellaceae	-0.090	-0.143	-0.043	-0.039	-0.108	0.034	**0.226**
	Lachnospiraceae	-0.027	0.005	-0.068	**0.214**	-0.025	**-0.212**	0.045
Genus	*Prevotella.1*	-0.230	0.220	-0.186	-0.299	0.145	0.412	0.045
	Escherichia	0.051	-0.117	0.035	0.030	-0.109	-0.088	0.102
	Faecalibacterium	-0.033	0.117	-0.055	0.130	0.102	-0.096	-0.115*
	Bacteroides	0.052	-0.172	-0.161	-0.589*	-0.016	0.292	**0.385**
	Megamonas	-0.168	-0.146	-0.408	-0.165	-0.201	-0.140	**0.681**

* indicates $P < 0.1$, **bold** indicates $P < 0.05$; All models were adjusted by age, gender, BMI, family income, region, smoking, drinking, physical activities, antibiotic use, diabetes, chronic kidney disease, hypertension, and the scores of five dietary patterns.

Table S8 The condPIPs for EMs associated with log denoised counts of individual bacterial taxa

Level	taxon	V	Co	Se	Sr	Mg	Ca	Mo
Phylum	*Firmicutes*	0.276	0.422	0.026	0.103	0.096	**0.801**	0.276
	Bacteroidetes	0.237	0.156	0.467	**0.552**	0.055	0.393	0.140
	Proteobacteria	0.278	0.235	0.099	0.337	0.346	0.317	0.388
	Actinobacteria	0.242	0.356	0.207	0.168	0.347	0.484	0.195
	Tenericutes	0.066	0.031	0.020	0.314	0.297	0.390	**0.882**
Class	*Clostridia*	-	-	-	-	-	-	-
	Bacteroidia	0.240	0.360	<0.001	**0.634**	0.170	0.195	0.400
	Gammaproteobacteria	-	-	-	-	-	-	-
	Bacilli	0.252	0.462	0.144	0.150	0.479	0.371	0.142
	Actinobacteria	0.211	0.471	0.147	0.160	**0.508**	0.332	0.170
Order	*Clostridiales*	0.015	**0.908**	<0.001	0.479	0.062	0.459	0.076
	Bacteroidales	0.193	0.163	0.139	**0.510**	0.126	0.365	**0.506**
	Enterobacteriales	<0.001	**1.000**	<0.001	**0.863**	0.098	0.039	<0.001
	RF39	0.346	0.311	0.082	0.311	0.271	0.418	0.261
	Bifidobacteriales	0.173	**0.773**	0.030	**0.566**	0.182	0.251	0.023
Family	*Ruminococcaceae*	0.067	**0.591**	0.023	0.325	0.138	**0.538**	0.318
	Prevotellaceae	0.192	0.212	0.471	**0.687**	0.041	0.272	0.125
	Enterobacteriaceae	**0.726**	0.142	0.081	0.110	0.161	0.729	0.051
	Veillonellaceae	0.331	0.107	**0.502**	**0.670**	0.077	0.253	0.060
	Lachnospiraceae	**0.573**	0.346	0.042	0.199	0.023	**0.778**	0.039
Genus	*Prevotella.1*	0.251	0.281	0.243	0.188	0.215	**0.597**	0.225
	Escherichia	0.308	0.419	0.099	**0.642**	0.080	0.278	0.175
	Faecalibacterium	0.064	**0.670**	0.012	0.394	0.157	0.450	0.254
	Bacteroides	0.231	0.142	0.151	**0.975**	0.007	0.018	0.476
	Megamonas	0.430	0.044	0.174	**0.746**	0.022	0.232	0.352

bold indicates PIP > 0.500; **-**, due to the too small difference among the abundance data, the BKMR models failed to fit normally; All models were adjusted by age, gender, BMI, family income, region, smoking, drinking, physical activities, antibiotic use, diabetes, chronic kidney disease, hypertension, and the scores of five dietary patterns.

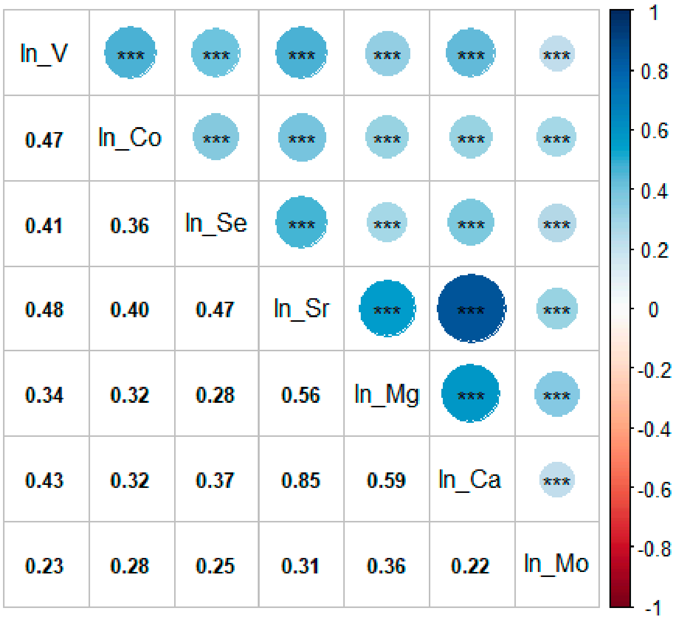

Figure S1 Pearson correlation of ln creatinine-adjusted urinary EMs concentration. *** indicates $P < 0.001$

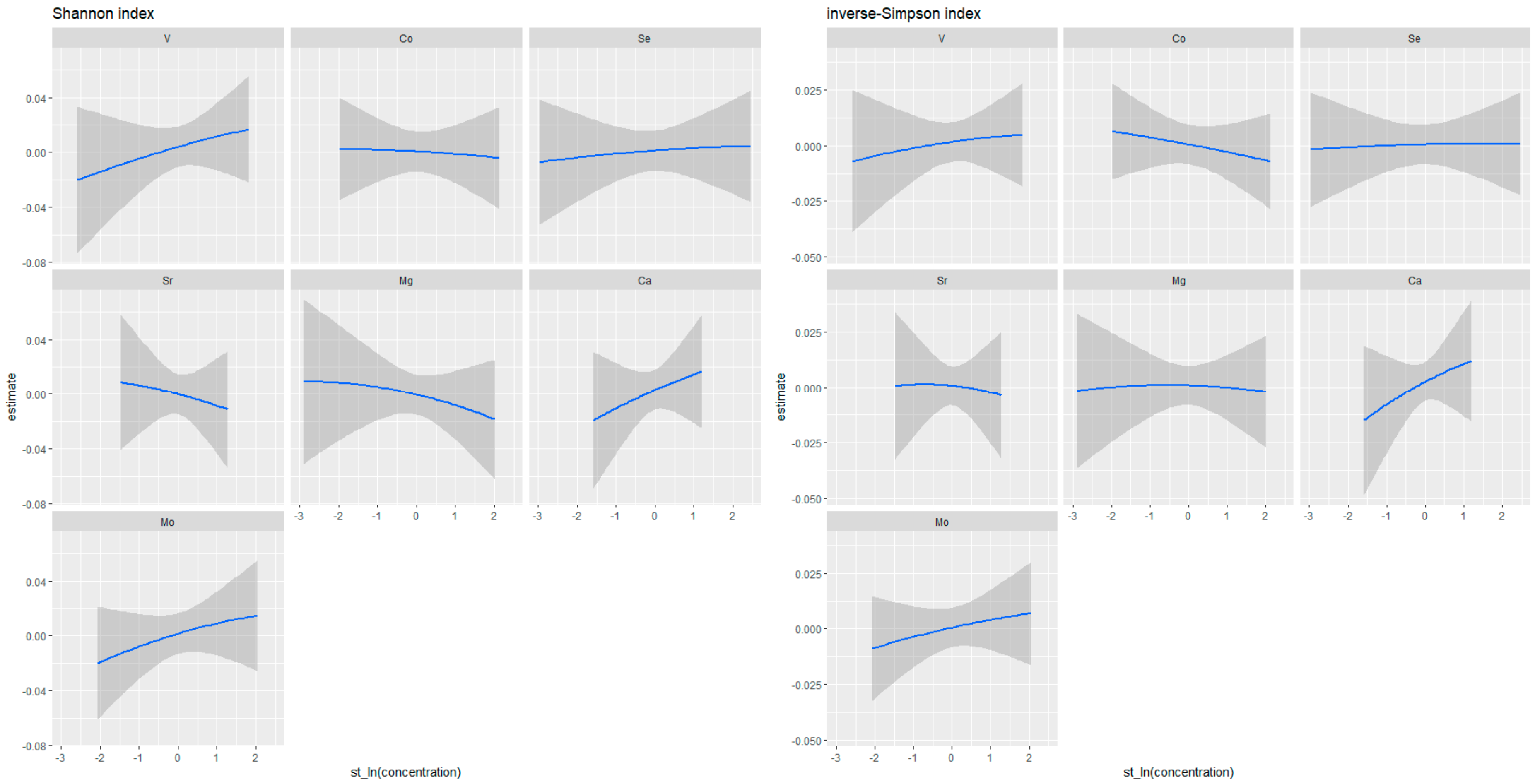

Figure S2 Univariate exposure response functions and 95% confidence intervals (CIs) for single EMs when all other exposures are fixed at the median

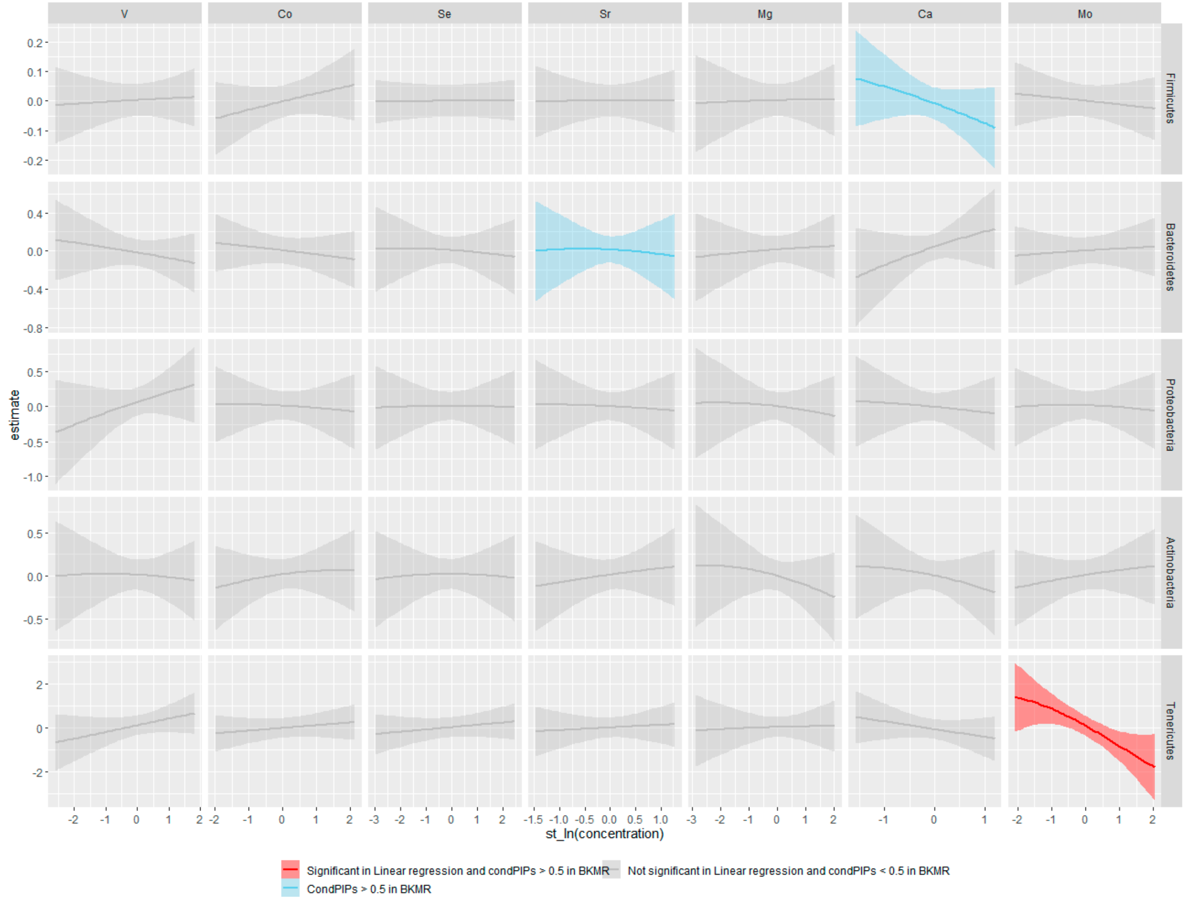

Figure S3 Univariate exposure response functions and 95% confidence intervals (CIs) for single EMs when all other exposures are fixed at the median, for the top 5 most abundant taxa at the phylum level. The Color indicates the significance in linear regressions or BKMR models. All models were adjusted by age, gender, BMI, family income, region, smoking, drinking, physical activities, antibiotic use, diabetes, chronic kidney disease, hypertension, and the scores of five dietary patterns.

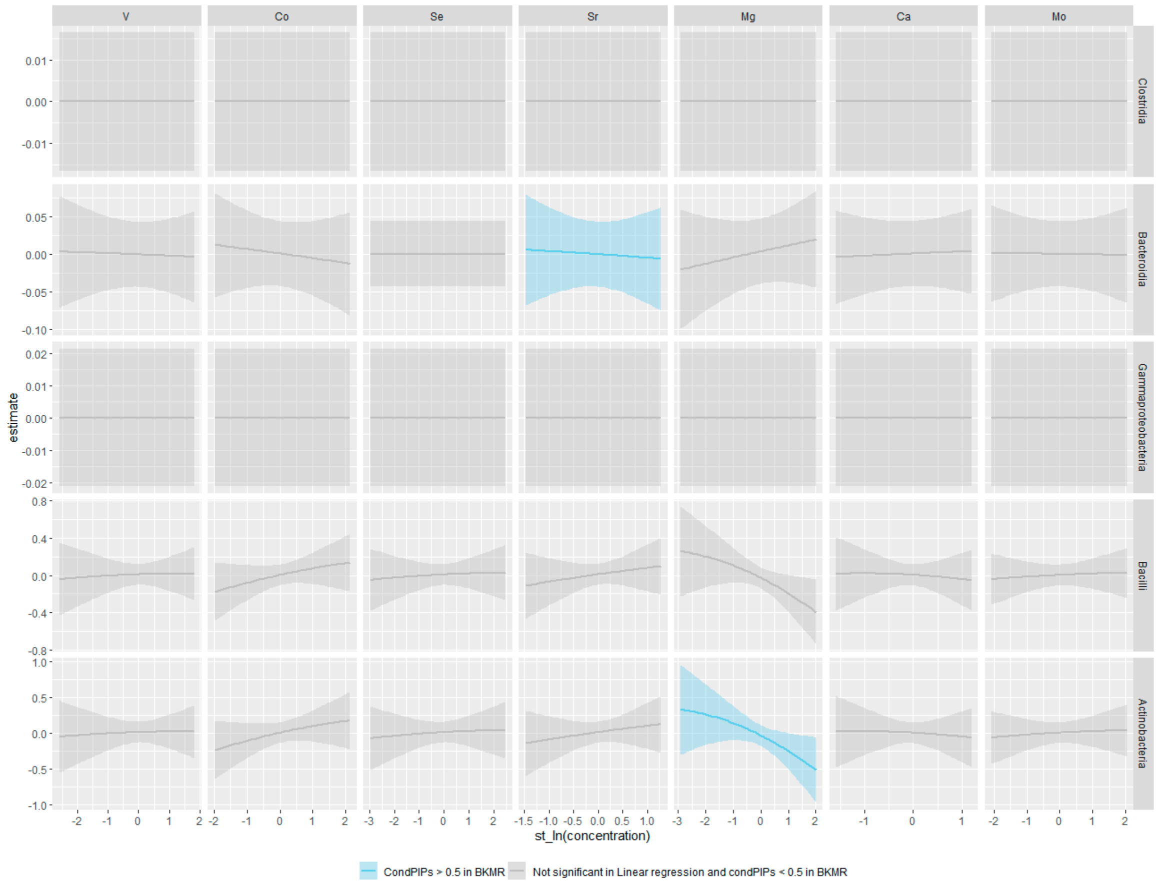

Figure S4 Univariate exposure response functions and 95% confidence intervals (CIs) for single EMs when all other exposures are fixed at the median, for the top 5 most abundant taxa at the class level. The Color indicates the significance in linear regressions or BKMR models. All models were adjusted by age, gender, BMI, family income, region, smoking, drinking, physical activities, antibiotic use, diabetes, chronic kidney disease, hypertension, and the scores of five dietary patterns.

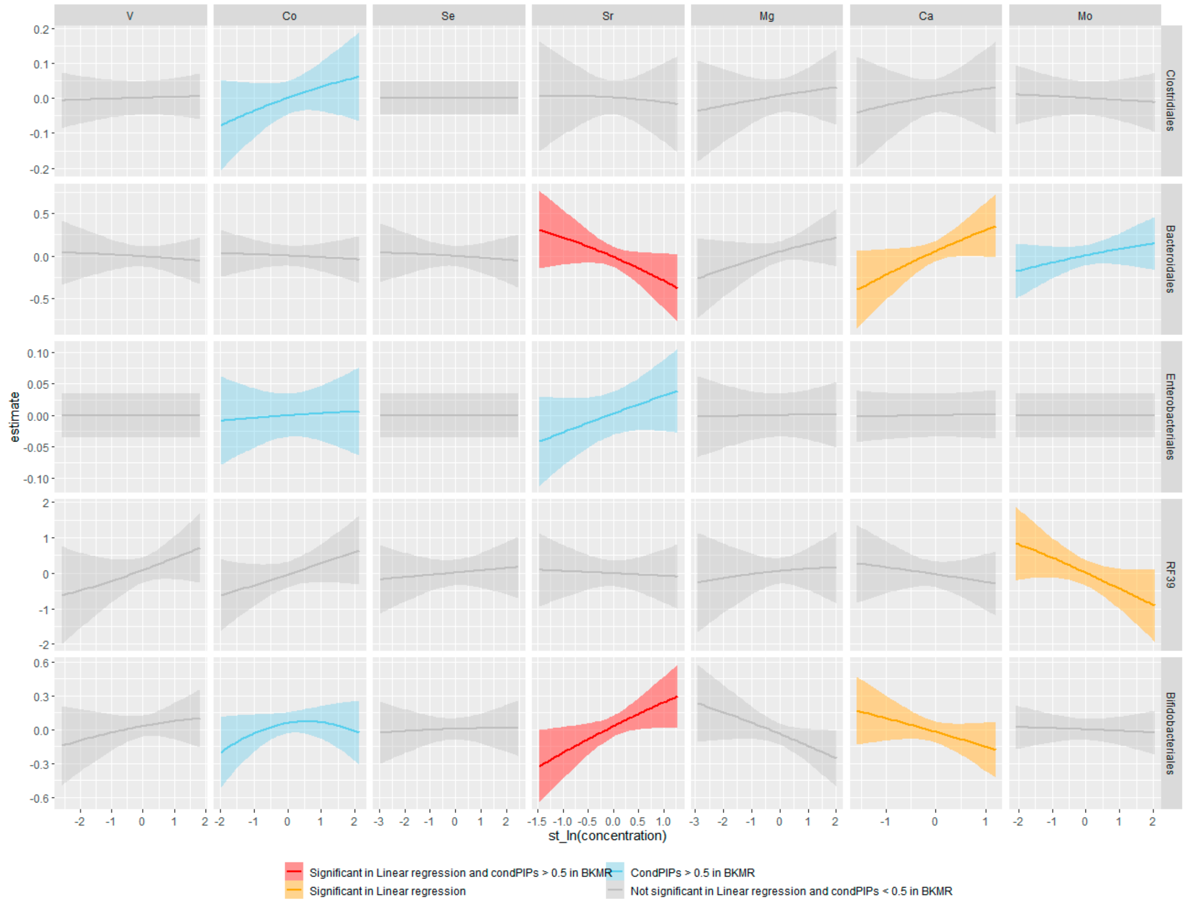

Figure S5 Univariate exposure response functions and 95% confidence intervals (CIs) for single EMs when all other exposures are fixed at the median, for the top 5 most abundant taxa at the order level. The Color indicates the significance in linear regressions or BKMR models. All models were adjusted by age, gender, BMI, family income, region, smoking, drinking, physical activities, antibiotic use, diabetes, chronic kidney disease, hypertension, and the scores of five dietary patterns.

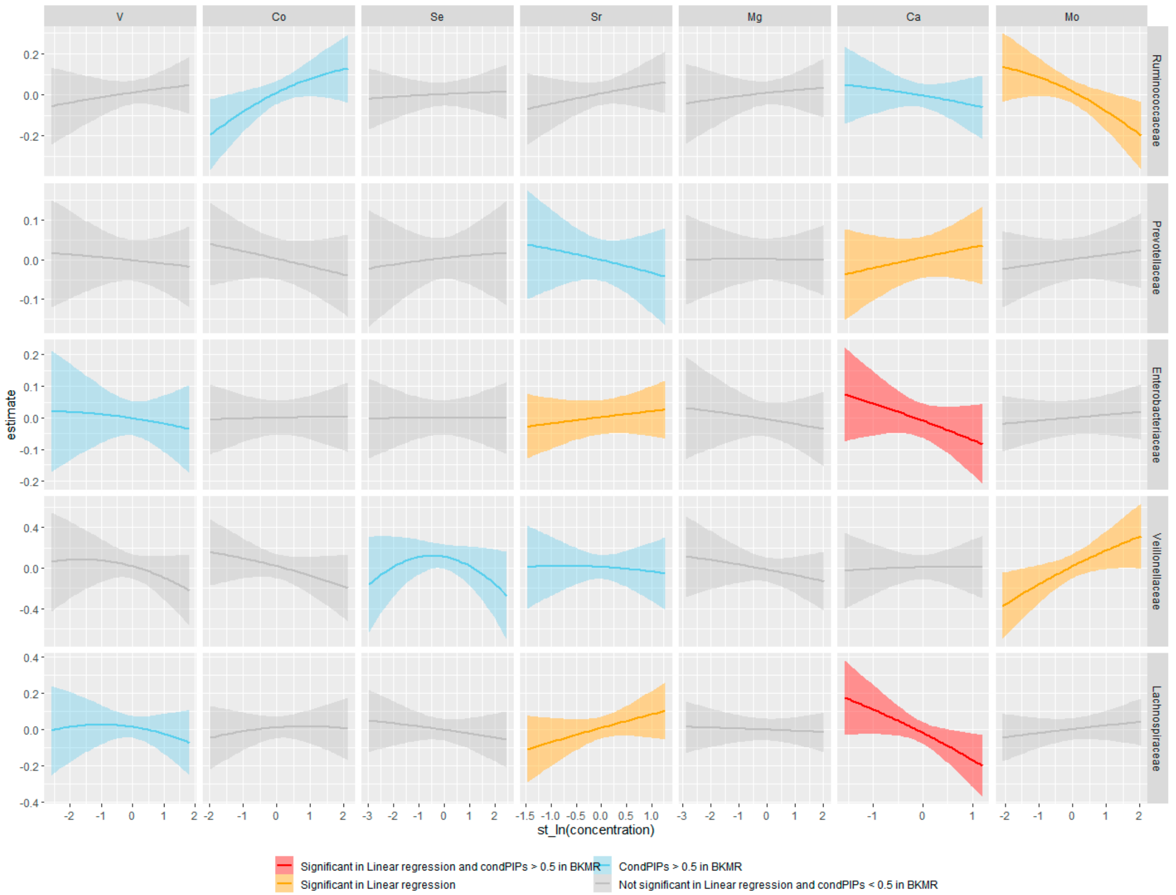

Figure S6 Univariate exposure response functions and 95% confidence intervals (CIs) for single EMs when all other exposures are fixed at the median, for the top 5 most abundant taxa at the family level. The Color indicates the significance in linear regressions or BKMR models. All models were adjusted by age, gender, BMI, family income, region, smoking, drinking, physical activities, antibiotic use, diabetes, chronic kidney disease, hypertension, and the scores of five dietary patterns.

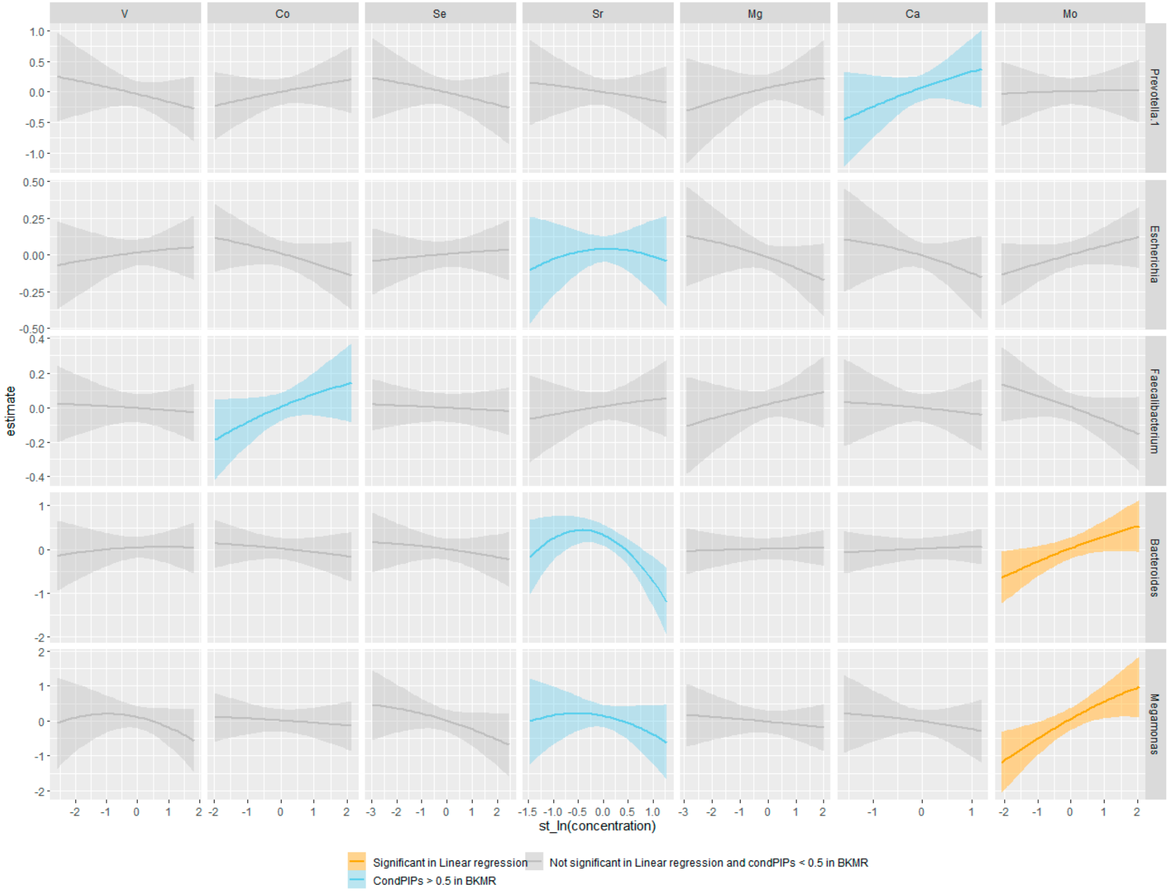

Figure S7 Univariate exposure response functions and 95% confidence intervals (CIs) for single EMs when all other exposures are fixed at the median, for the top 5 most abundant taxa at the genus level. The Color indicates the significance in linear regressions or BKMR models. All models were adjusted by age, gender, BMI, family income, region, smoking, drinking, physical activities, antibiotic use, diabetes, chronic kidney disease, hypertension, and the scores of five dietary patterns.

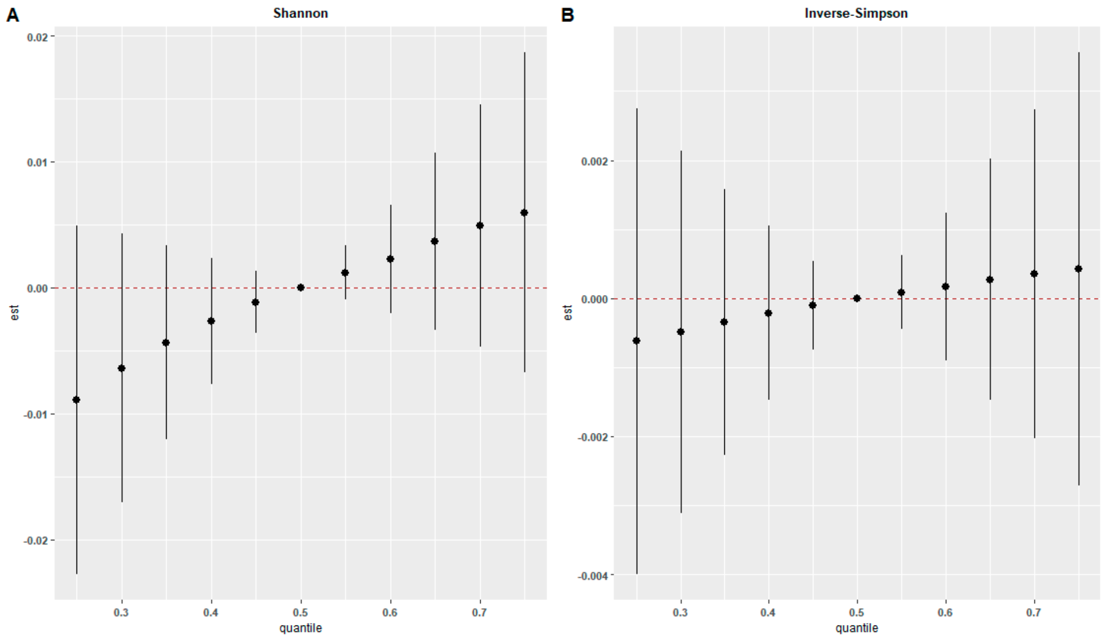

Figure S8 Joint exposure effect of the mixture (95% CIs) for Shannon index (A) and Inverse-Simpson index (B), defined as the predicted change in the outcome when all EMs were held at particular percentiles, compared to when all metals were held at their median concentration.

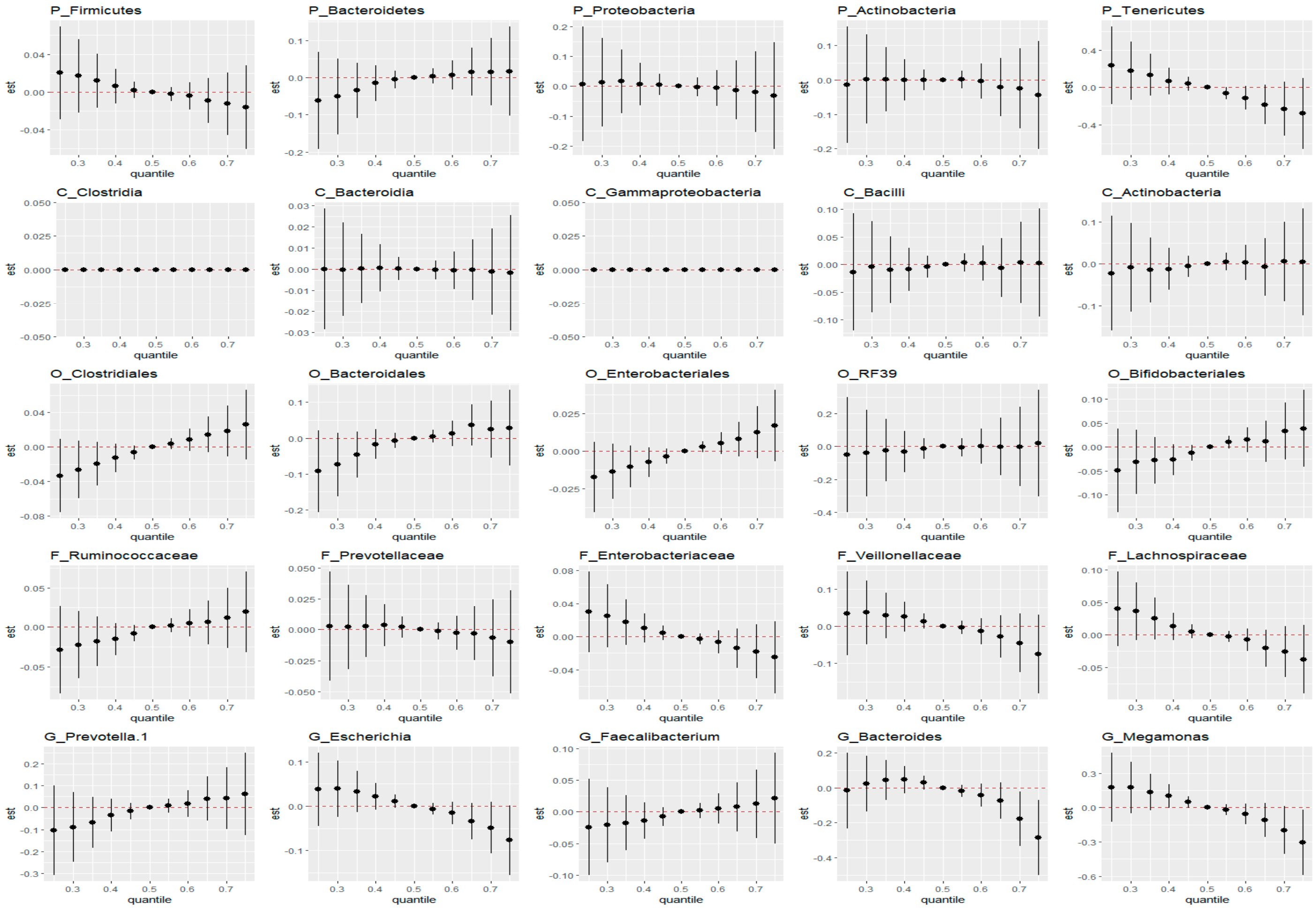

Figure S9. Joint exposure effect of the mixture (95% CIs) for all selected taxa, defined as the predicted change in the outcome when all EMs were held at particular percentiles, compared to when all metals were held at their median concentration.

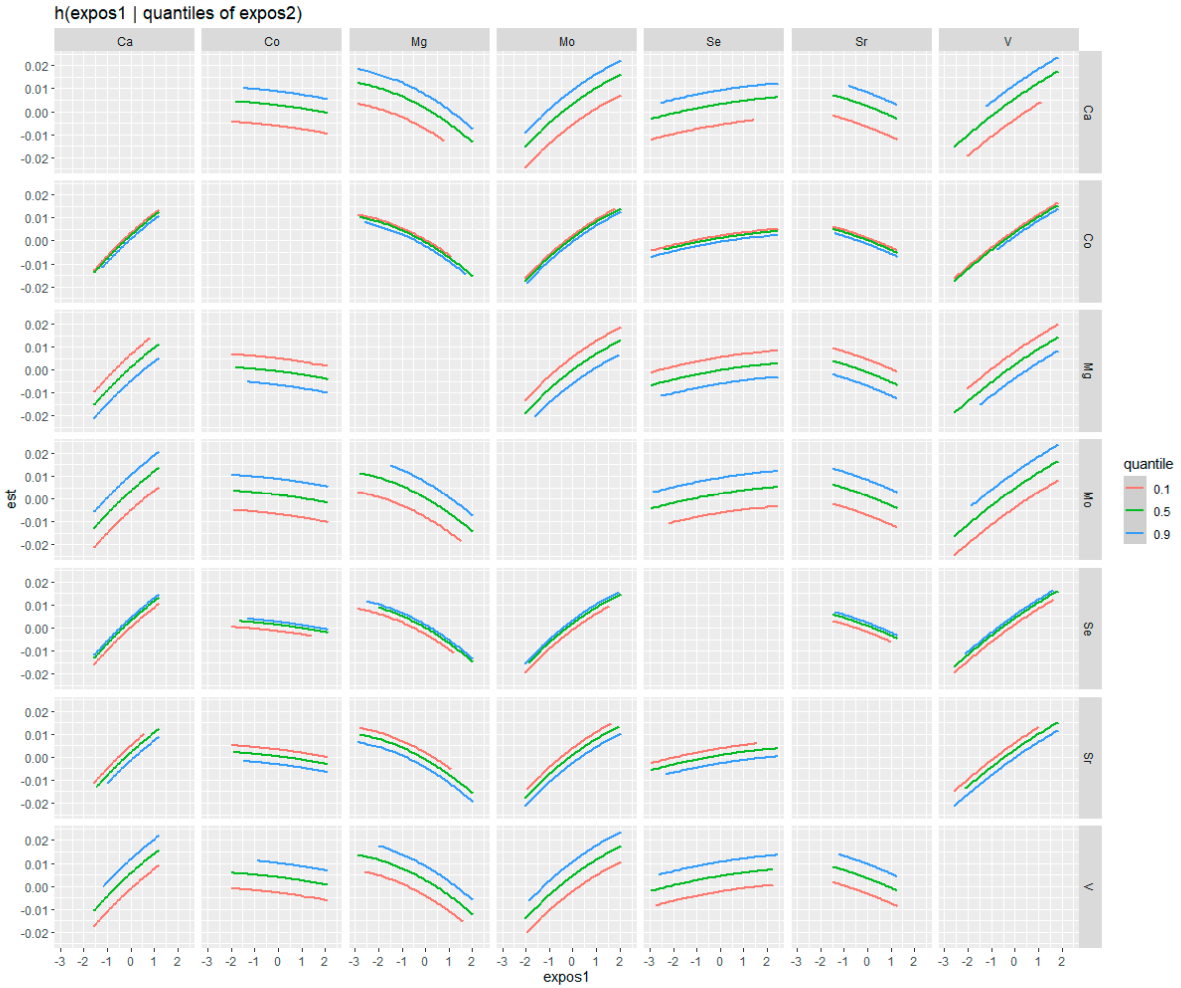

Figure S10 Bivariate exposure response functions for Shannon index. Each cell represented the exposure-response curve for the column EM when the row EM was fixed at 10th, 50th, and 90th percentiles and the remaining EMs were fixed at their medians.

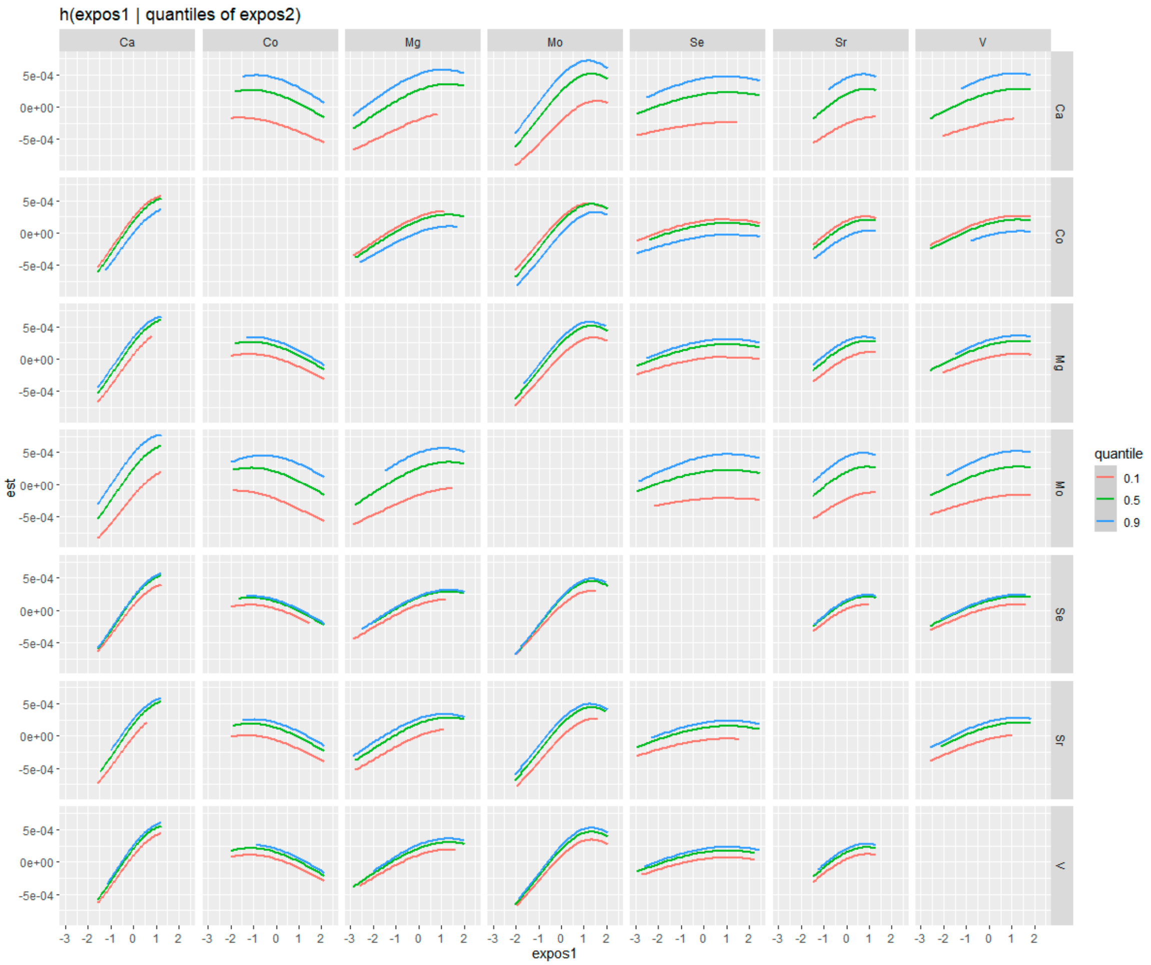

Figure S11 Bivariate exposure response functions for Inverse-Simpson index. Each cell represented the exposure-response curve for the column EM when the row EM was fixed at 10th, 50th, and 90th percentiles and the remaining EMs were fixed at their medians.

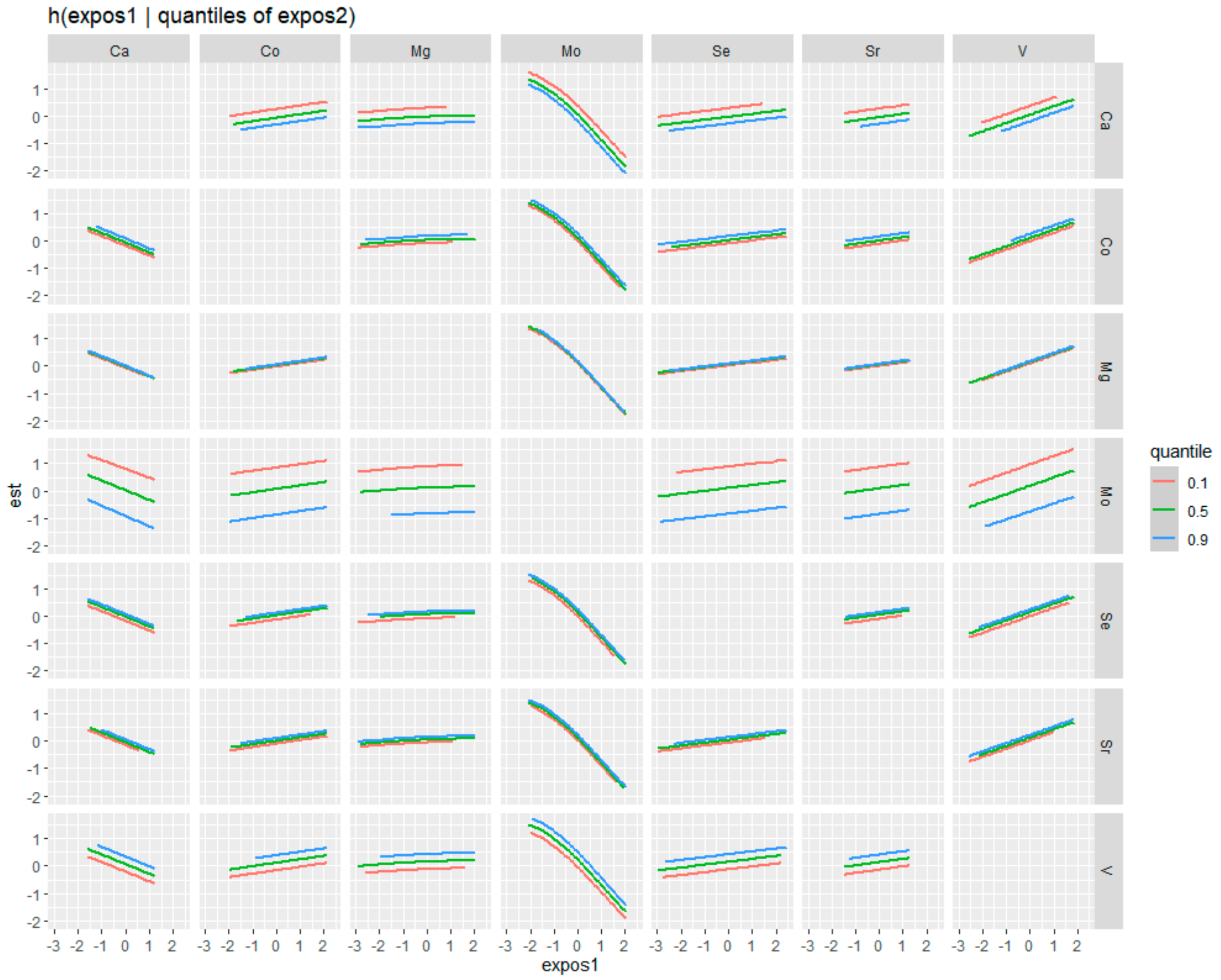

Figure S12 Bivariate exposure response functions for *Tenericutes*. Each cell represented the exposure-response curve for the column EM when the row EM was fixed at 10th, 50th, and 90th percentiles and the remaining EMs were fixed at their medians.

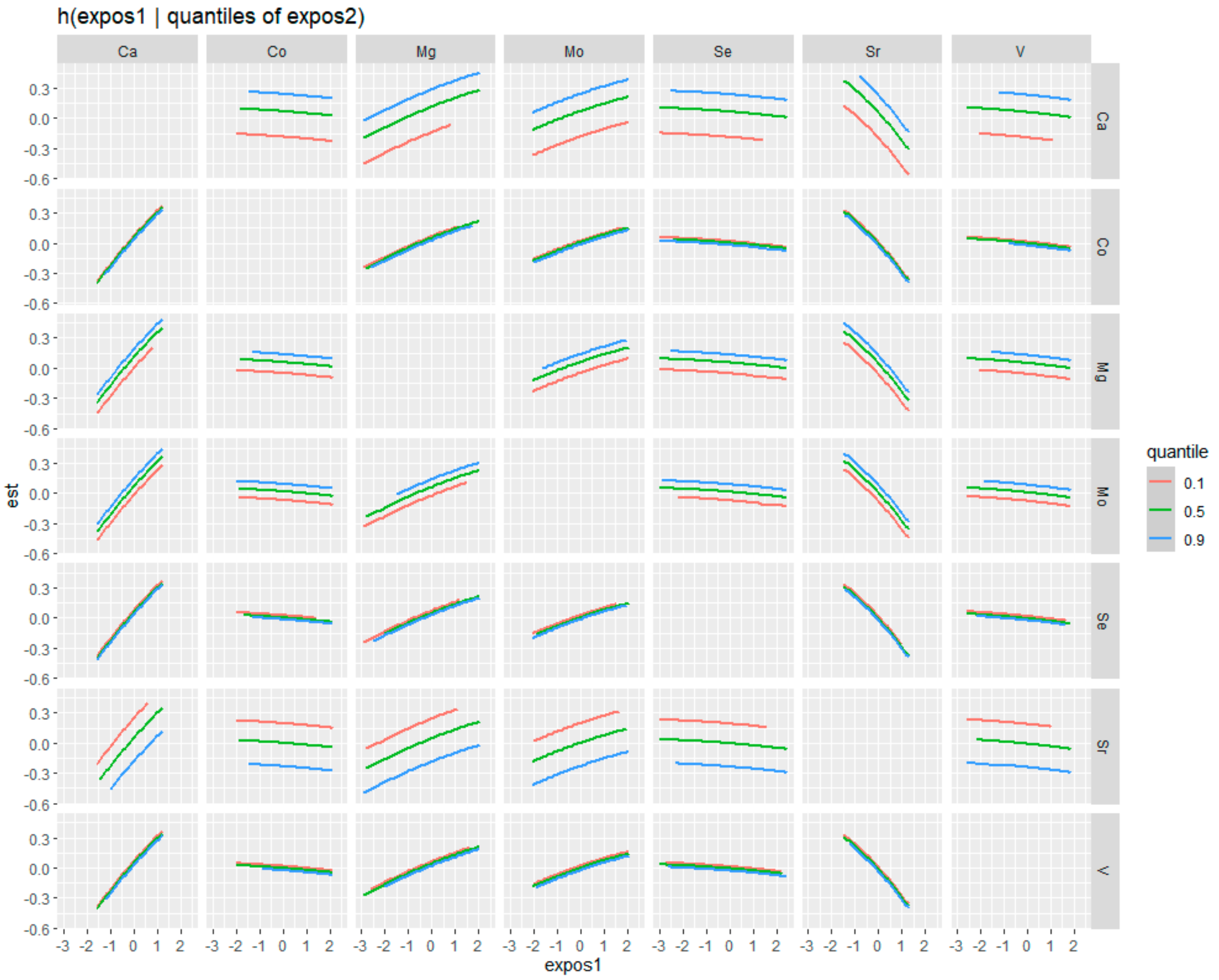

Figure S13 Bivariate exposure response functions for *Bacteroidales.* Each cell represented the exposure-response curve for the column EM when the row EM was fixed at 10th, 50th, and 90th percentiles and the remaining EMs were fixed at their medians.

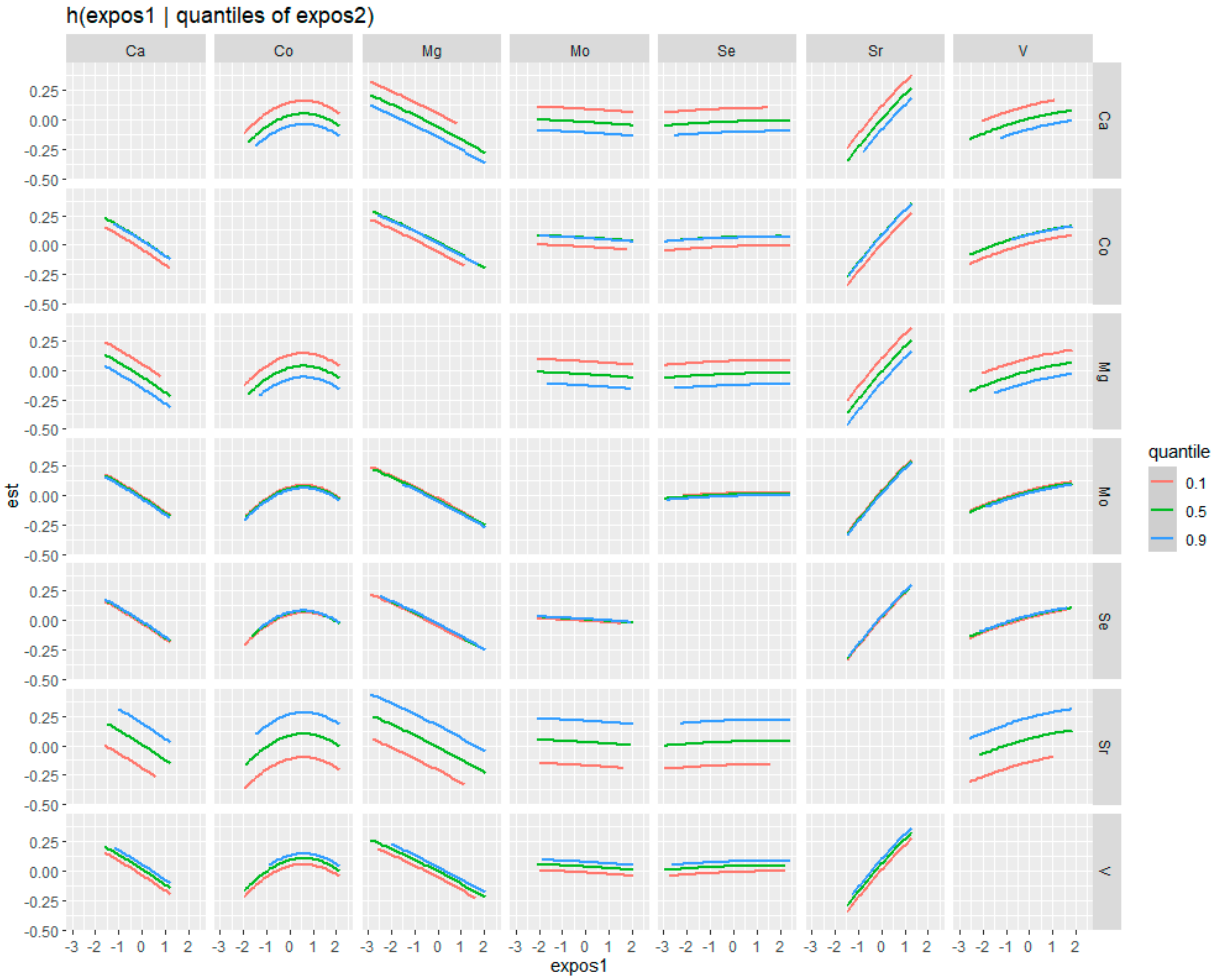

Figure S14 Bivariate exposure response functions for *Bifidobacteriales*. Each cell represented the exposure-response curve for the column EM when the row EM was fixed at 10th, 50th, and 90th percentiles and the remaining EMs were fixed at their medians.

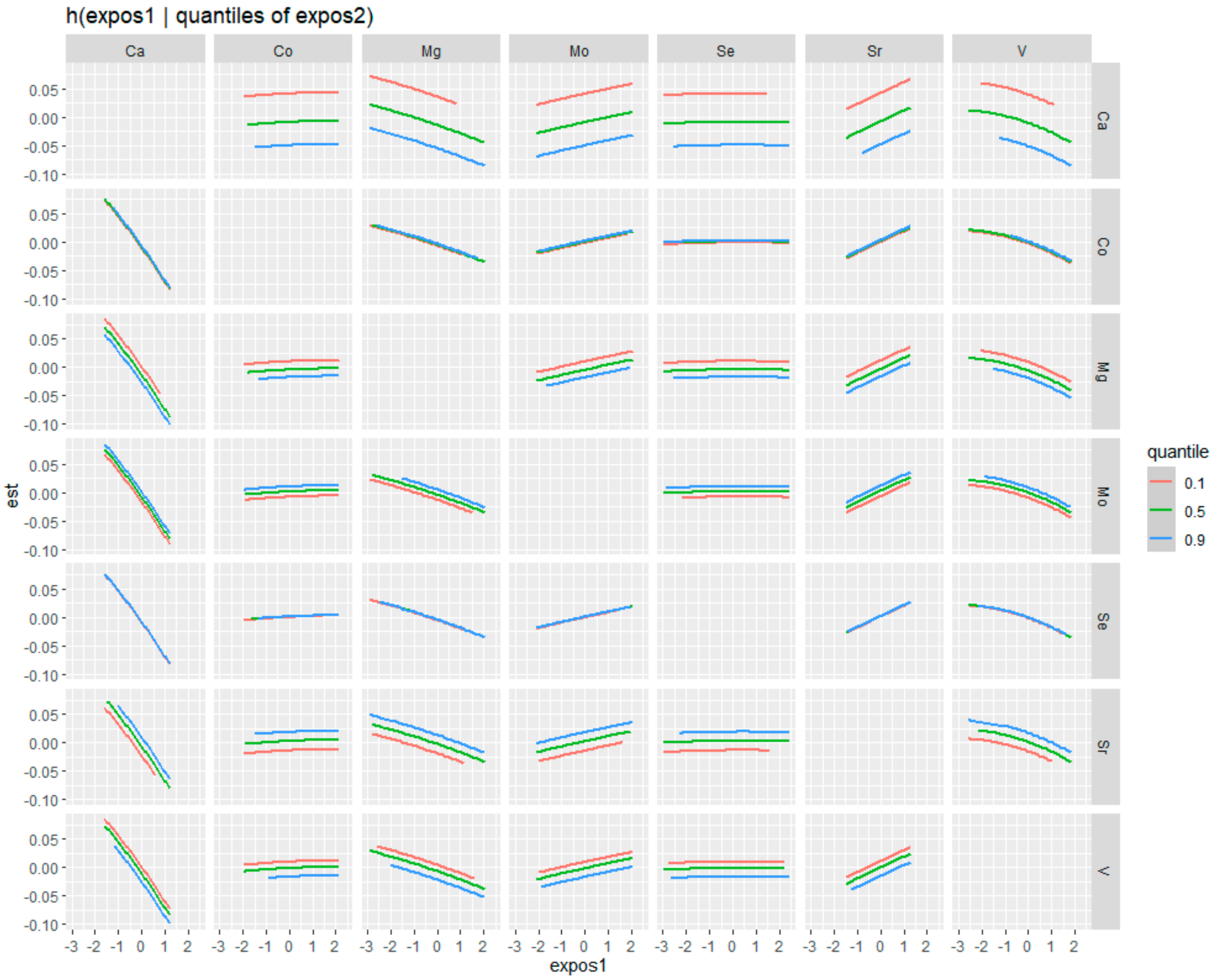

Figure S15 Bivariate exposure response functions for *Enterobacteriaceae*. Each cell represented the exposure-response curve for the column EM when the row EM was fixed at 10th, 50th, and 90th percentiles and the remaining EMs were fixed at their medians.

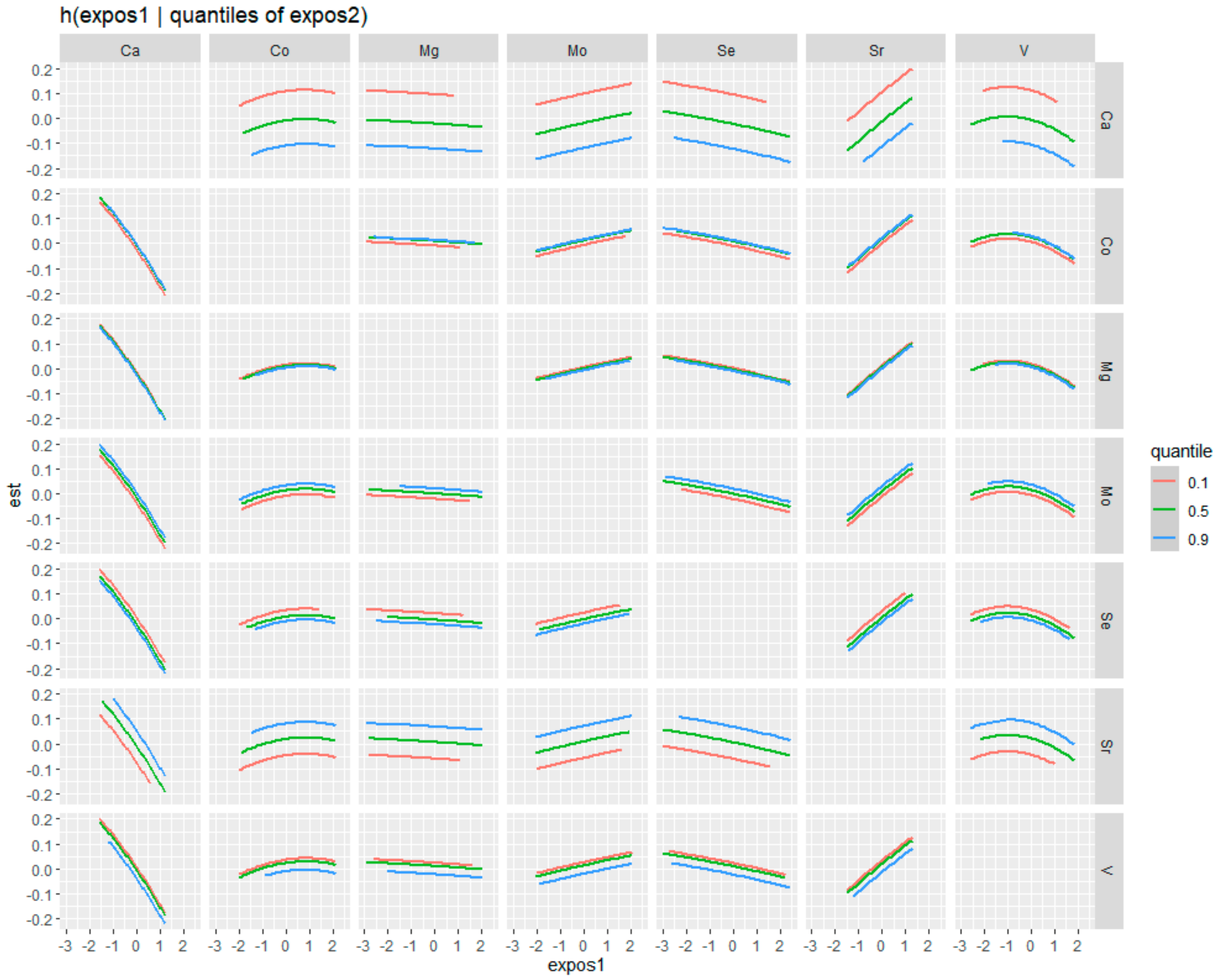

Figure S16 Bivariate exposure response functions for *Lachnospiraceae*. Each cell represented the exposure-response curve for the column EM when the row EM was fixed at 10th, 50th, and 90th percentiles and the remaining EMs were fixed at their medians.

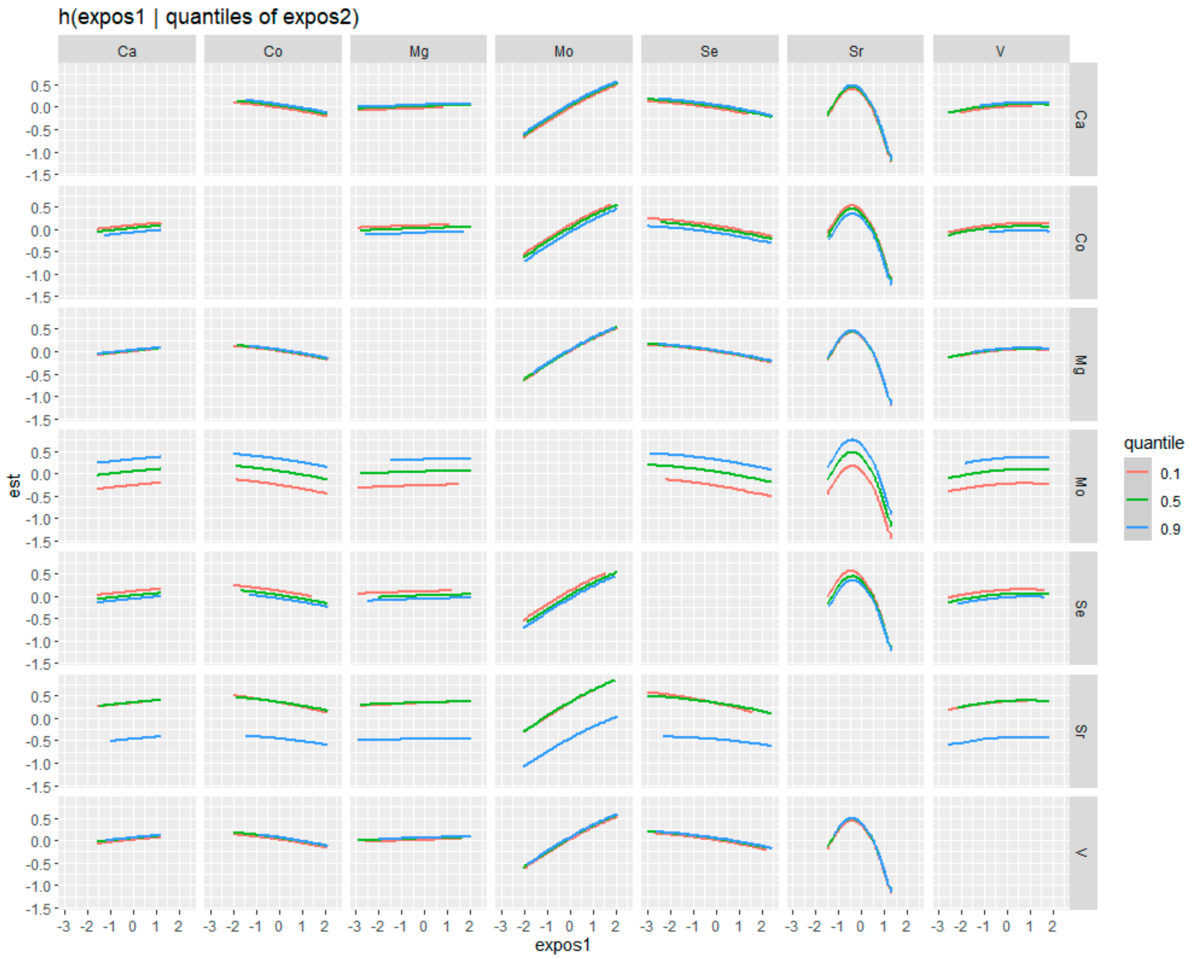

Figure S17 Bivariate exposure response functions for *Bacteroides*. Each cell represented the exposure-response curve for the column EM when the row EM was fixed at 10th, 50th, and 90th percentiles and the remaining EMs were fixed at their medians.

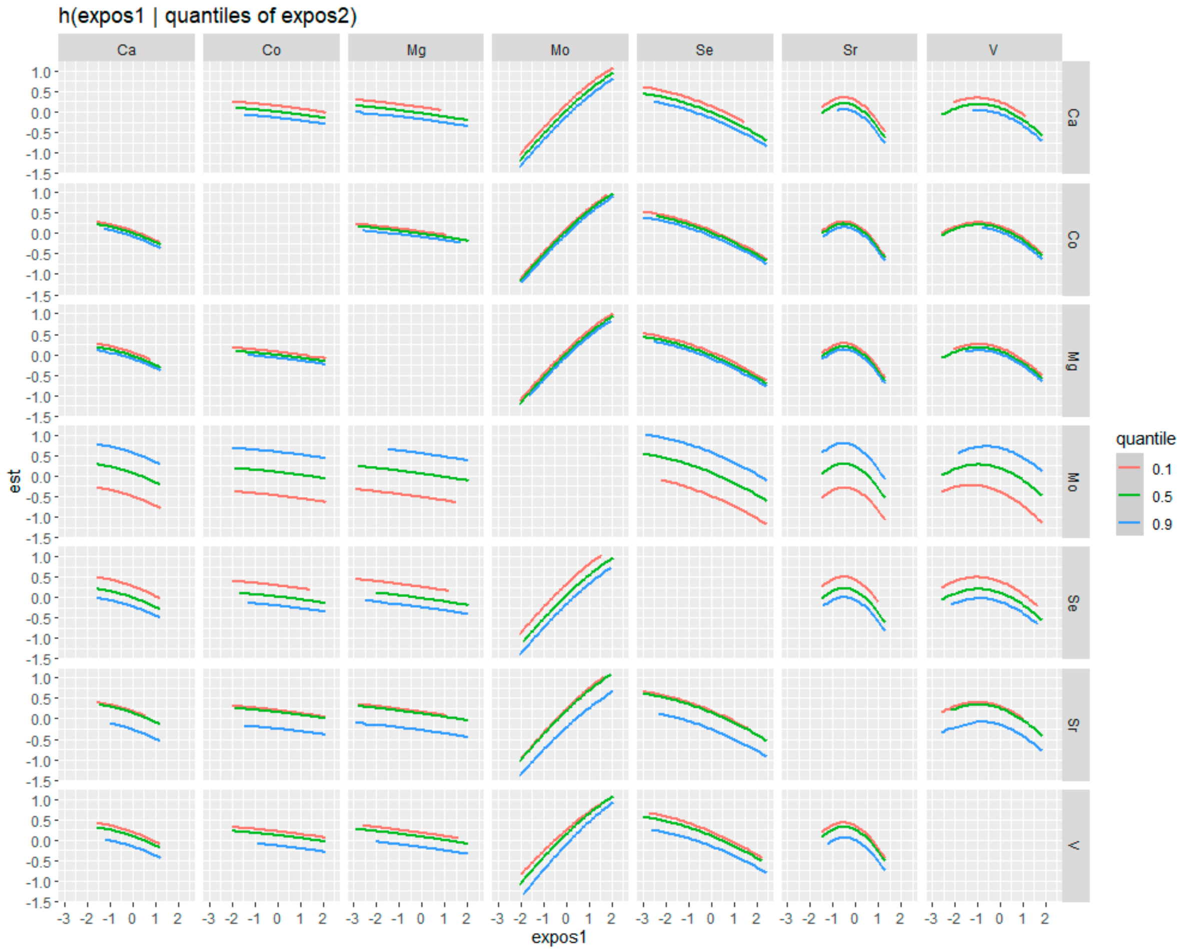

Figure S18 Bivariate exposure response functions for *Megamonas*. Each cell represented the exposure-response curve for the column EM when the row EM was fixed at 10th, 50th, and 90th percentiles and the remaining EMs were fixed at their medians.
